# ODE Constrained Mixture Modelling: A Method for Unraveling Subpopulation Structures and Dynamics

**DOI:** 10.1371/journal.pcbi.1003686

**Published:** 2014-07-03

**Authors:** Jan Hasenauer, Christine Hasenauer, Tim Hucho, Fabian J. Theis

**Affiliations:** 1 Institute of Computational Biology, Helmholtz Center Munich, Munich, Germany; 2 Division of Mathematical Modeling of Biological Systems, Department of Mathematics, University of Technology Munich, Munich, Germany; 3 Max Planck Institute for Molecular Genetics, Berlin, Germany; 4 Division of Experimental Anesthesiology and Pain Research, Department of Anesthesiology and Intensive Care Medicine, University Hospital Cologne, Cologne, Germany; ETH Zurich, Switzerland

## Abstract

Functional cell-to-cell variability is ubiquitous in multicellular organisms as well as bacterial populations. Even genetically identical cells of the same cell type can respond differently to identical stimuli. Methods have been developed to analyse heterogeneous populations, e.g., mixture models and stochastic population models. The available methods are, however, either incapable of simultaneously analysing different experimental conditions or are computationally demanding and difficult to apply. Furthermore, they do not account for biological information available in the literature. To overcome disadvantages of existing methods, we combine mixture models and ordinary differential equation (ODE) models. The ODE models provide a mechanistic description of the underlying processes while mixture models provide an easy way to capture variability. In a simulation study, we show that the class of ODE constrained mixture models can unravel the subpopulation structure and determine the sources of cell-to-cell variability. In addition, the method provides reliable estimates for kinetic rates and subpopulation characteristics. We use ODE constrained mixture modelling to study NGF-induced Erk1/2 phosphorylation in primary sensory neurones, a process relevant in inflammatory and neuropathic pain. We propose a mechanistic pathway model for this process and reconstructed static and dynamical subpopulation characteristics across experimental conditions. We validate the model predictions experimentally, which verifies the capabilities of ODE constrained mixture models. These results illustrate that ODE constrained mixture models can reveal novel mechanistic insights and possess a high sensitivity.

## Author Summary

In this manuscript, we introduce ODE constrained mixture models for the analysis of population snapshot data of kinetics and dose responses. Population snapshot data can for instance be derived from flow cytometry or single-cell microscopy and provide information about the population structure and the dynamics of subpopulations. Currently available methods enable, however, only the extraction of this information if the subpopulations are very different. By combining pathway-specific ODE and mixture models, a more sensitive method is obtained, which can simultaneously analyse a variety of experimental conditions. ODE constrained mixture models facilitate the reconstruction of subpopulation sizes and dynamics, even in situations where the subpopulations are hardly distinguishable. This is shown for a simulation example as well as for the process of NGF-induced Erk1/2 phosphorylation in primary sensory neurones. We find that the proposed method allows for a simple but pervasive analysis of heterogeneous cell systems and more profound, mechanistic insights.

This is a *PLOS Computational Biology* Methods article.

## Introduction

Multi-cellular organisms are faced with diverse, ever changing environments. To ensure survival and evolutionary success, microbial systems exploit cell-to-cell variability originating from bet-hedging strategies which increase the robustness against environmental changes [1]. Such bet-hedging relies on the formation of cellular subpopulations with distinct phenotypes and has been observed in the context of food source selection [2] and cellular stress response [3]. More complex organisms, such as mammals, evolved strategies to actively detect and respond to environmental changes. The building blocks for the necessary structures and functional units are cell types with distinct properties [4]. These cell types, e.g., neurones and immune cells, split up in further cellular subpopulations – cluster of cells with similar properties – to allow for a fine-grained recognition and tailored response. Due to the ubiquity of structured population heterogeneity, the analysis of subpopulation characteristics and causal differences between subpopulations is crucial for a holistic understanding of biological processes.

**Figure 1:**
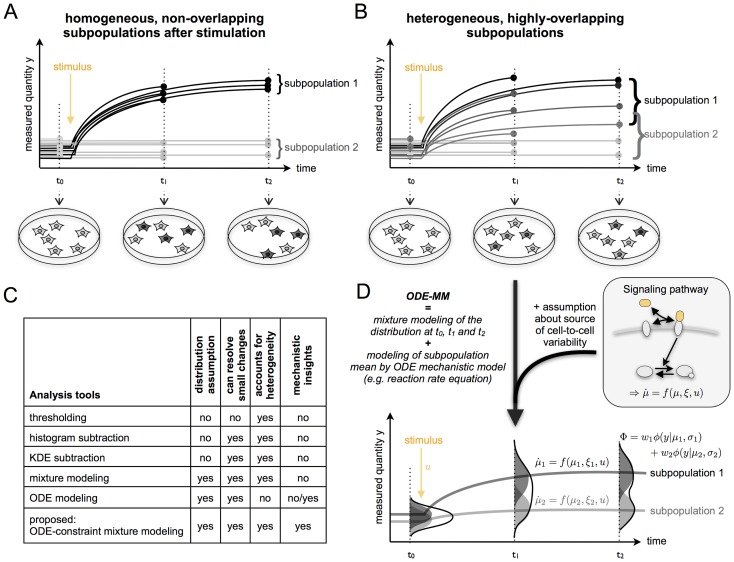
Illustration of population snapshot data and ODE constrained mixture modelling. (A) Heterogeneous population consisting of two homogeneous subpopulations with a very different response level. Snapshot data provide at different time points (filled circle) information about the biological state of single cells. This allows for the characterisation of the kinetics of the subpopulations using threshold, histogram and kernel density estimate (KDE) based methods as well as mixture modelling. (B) Heterogeneous population consisting of two heterogenous subpopulations with a large overlap of the dose response behaviour, rendering an analysis using snapshot data difficult. (C) Table including the available analysis tools for population snapshot data and proposed ODE constrained mixture modelling along with key properties of the methods. (D) Sketch of ODE constrained mixture modelling which combines mixture modelling of the measurement data with pathway information, thereby allowing for an improved quantification of subpopulation properties and mechanistic insights.

Heterogeneous cell populations are usually investigated using molecular and cell-biological methods with single cell resolution. Currently available methods include microscopy [5], [6], flow cytometry [7], single-cell PCR [8]–[10] and single-cell mass spectrometry [11]. While some microscopy based approaches provide possibly time-resolved data [5], most experimental techniques do not allow for the tracking of individual cells but provide snapshots of the population. In this study, we considered these snapshot data, which can provide information about cellular properties, such as protein expression and phosphorylation. An illustration of snapshot data is provided in Figures 1A and B.

The analysis of population snapshot data can be approached using a multitude of statistical methods, e.g., thresholding, density based methods and mixture modelling. The selection of the method is highly problem specific [12]. Thresholding methods are the most commonly used tools to identify the size of a subpopulation, e.g., the size of a subpopulation expressing a particular marker [13]. Based on a control experiment a threshold (or gate) is defined based on which cells are classified as marker positive or negative. While thresholding works in cases of clearly separated subpopulations (Figure 1A), it fails for strongly overlapping heterogeneous populations (Figure 1B) as no appropriate threshold exists, resulting in large numbers of false positives and/or false negatives. Furthermore, thresholding only detects large changes rendering it insensitive. An improved sensitivity is achieved by density based methods, namely histogram-based and kernel density estimation (KDE)-based methods [14]–[19], which compare the full distributions. Nevertheless, also density based methods tend to underestimate the size of positive/responsive subpopulations. This is not the case for mixture models which describe the cell population as a weighted sum of the underlying subpopulations. The underlying subpopulations are described using simple distributions functions [7], [20]–[22], those statistical properties, e.g., mean and variance, describe the subpopulation. The outcome of a mixture model based data analysis depends, more or less sensitively, on the distribution assumption [7]. To assess the temporal evolution of subpopulations, matching is performed [7], [12].

In addition to the aforementioned shortcoming, currently available statistical methods can only analyse measured snapshot data. None of the methods provides directly mechanistic insights, prediction for hidden network components, hypotheses regarding causal factors for the population heterogeneity or estimates for reaction rates. To gain such additional insight and to simultaneously analyse multiple snapshots, a mechanistic description of the underlying process is required. Mostly, such descriptions are based on ordinary differential equations (ODEs). Commonly used ODE models, however, do not allow for the integration of distributional information but only use the measured mean concentration [23]–[26]. A summary of data analysis tools and their key properties is provided in Figure 1C.

In the following, we propose ODE constrained mixture models (ODE-MMs), a combination of mixture models and ODE based pathway models which exploits their individual advantages (Figure 1D). This novel class of models describes the individual snapshots using mixtures whose components are constrained by ODE models. These ODE models for the subpopulations are derived from the pathway topology and assumptions about causal, mechanistic differences between subpopulations. Due to the underlying mechanistic description of subpopulation dynamics, ODE-MMs can go beyond the obvious. Instead of only analysing the measured quantities and performing error-prone matching across conditions across multiple snapshots, ODE-MMs are capable of determining the dynamics of hidden components and testing for causal differences between subpopulations. This is illustrated using a simulation study of a conversion process.

Exemplarily, ODE-MMs are applied to investigate NGF-induced Erk1/2 phosphorylation in primary sensory neurones, a signalling pathway regulating pain sensitisation. Due to the diverse functional roles of sensory neurones, the cell system is highly heterogeneous. We introduce a dynamical model for NGF-induced Erk1/2 phosphorylation in primary sensory neurones and attempt the unraveling of the subpopulation structure and the source of heterogeneity using ODE-MMs. The results are validated using co-labelling experiments.

## Methods

### Ethics statement

All animal experiments were reported to the responsible authority, the *Landesamtes für Gesundheit und Soziales* (LAGeSo) in Berlin (T0370/05) and approved (license ZH120). All efforts were made to minimise the number of animals used and their suffering.

### Measurement data

In this work we consider collections 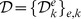 of population snapshot data 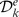, as illustrated in Figures 1A and B. Experimental conditions are indexed by 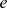 and time points are indexed by 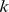. The individual snapshots 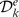 are measured at time 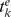 under experimental condition 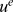. 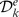 is a collection of single cell measurements 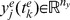, 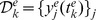, with 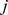 indexing the individual cells. The single cell measurements are assumed to be statistically independent.

### Mixture models

The analysis of the individual population snapshots 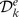, which are samples of cells, is often approached using mixture models,
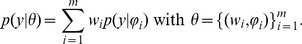
(1)Parameters and probability weights of the 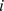-th mixture component are denoted by 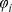 and 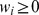, with 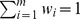, respectively. Common choices for the individual mixture components 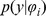 are normal, log-normal, skew normal, t-, and skew t-distributions [7]. In the case of normal mixtures the component parameters are mean 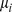 and covariance matrix 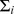, 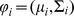. The parameters 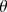 of mixture models can be estimated using maximum likelihood methods,
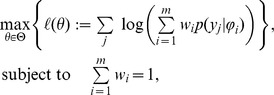
in which 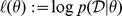 denotes the log-likelihood function of the mixture model and 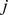 is the index of the single cell measurement. The set of possible parameter values is denoted by 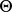.

The individual mixture components are often regarded as subpopulations with different characteristics, e.g., different expression levels. To analyse collections of snapshots 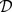, a matching of subpopulations detected under different conditions is performed [7], [12]. The results of this matching can in principle be used to extract the characteristics of subpopulations and their dependence on time and stimuli. The matching performed between individual conditions is however often questionable, in particular if some populations change their characteristics dramatically or are not/hardly distinguishable under some condition. In this case matching-based methods are highly error-prone [12].

### Pathway models

To circumvent shortcomings of mixture modelling, we propose to complement it with pathway information. The responses of subpopulations to different experimental conditions is ultimately determined by the involved metabolic, signalling and gene regulatory pathways. Accordingly, experimental conditions can be matched using models of the underlying biochemical pathway.

Biochemical pathways are mostly modelled using reaction rate equations (RREs) [24], which are systems of ODEs. RREs describe the temporal evolution of the “average state” of cells in a cell population, e.g., the abundance of signalling molecules and their activity, assuming that the population is homogeneous. More precisely, RREs implicitly assume that the variance in the abundance of chemical species across cells is small. Therefore, these models can neither be used to process the distributional information encoded in snapshot data nor to study cellular subpopulations.

While RRE based modelling of heterogenous cell populations consisting of different subpopulations is not desirable, RREs might be used to model the dynamics of rather homogeneous subpopulations. In the following, we will describe the “average dynamics” of cells in the 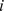-th subpopulation using a RRE,
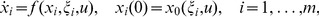
(2)in which 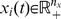 is the state of the 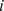-th subpopulation at time 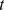, 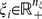 is the parameter vector of the 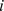-th subpopulation, and 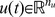 is the time-dependent external stimulus. The vector field 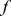 encodes the biochemical pathway and 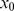 models the dependence of the initial condition on subpopulation parameters and experimental conditions. The subpopulation parameters 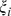 are a collection of parameters 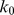 which are identical in all subpopulations and subpopulation specific parameters 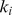, 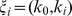. Identical parameters might be structural properties, such as affinities. Differences between subpopulations are modelled by differences in their parameters, 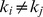. These parameter discrepancies describe the causal differences between subpopulations, e.g., altered protein abundances, and are biologically essential when studying heterogeneity.

As most experimental procedures only allow for the assessment of a few chemical species, we introduce a measurement model,
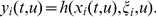
(3)If merely the 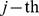 chemical species is observed this mapping becomes: 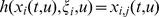.

Assuming that the communication across and transitions between subpopulations can be neglected for the process of interest, the dynamics of the overall population are captured by the weighted dynamics of its subpopulations. This idea is exploited by ODE-MMs, and will in the following be illustrated for mixtures of normal distributions and more general mixture distributions.

### RRE constrained mixture of normal distributions

The most commonly used mixture models are mixtures of normal distributions, 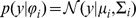, which are parameterised by mean 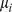 and covariance 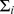. As RREs describe the dynamics of the mean state 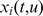 of homogeneous subpopulations, an obvious possibility is to model the condition- and time-dependent measured mean 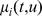 of the mixture components by RREs,
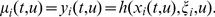
(4)Accordingly, the component means (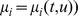 are determined by the parameters of the subpopulation 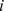, 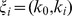. The component covariances (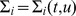), which summarise cell-to-cell variability within the 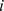-th subpopulation and measurement noise, are not constrained by RREs. Accordingly, we obtain RRE constrained mixture of normal distributions,
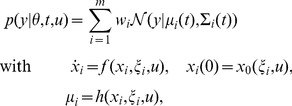
(5)with parameters 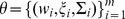. The mixture parameters, 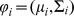, depend on experimental condition 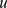 and time 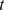. Furthermore, 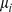 depends implicitly on 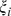 via the ODE model.

In contrast to conventional mixture models (1), ODE-MMs (5) describe the distribution of the observed variables at discrete points and the temporal evolution of subpopulations in response to stimuli. Hence, ODE-MMs establish a mechanistic link between different experimental conditions and time points based on pathway models and differences between subpopulations. This renders error-prone matching of distributions across conditions unnecessary (see discussion in *Mixture models*).

### General class of ODE constrained mixture models

The combination of normal mixture models and RRE models yields simple ODE-MMs. More flexible ODE-MMs are obtained by considering other distributions 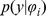, e.g., log-normal, skew normal, t- or skew t-distributions [7]. Furthermore, more sophisticated descriptions of the biochemical processes can be employed, e.g., linear noise approximations [27], [28], effective mesoscopic rate equations [29], [30] or moment equations [31], [32]. These classes of ODE models, 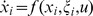, do not only constrained means but also variances, covariances and higher order moments. Hence, more distribution parameters 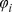 can be linked to the state of the ODE model, 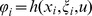. In general, the subpopulation parameters 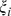 contain mechanistic parameters as well as parameters 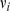 which specify statistics of the distribution, 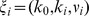.

### Parameter estimation and model selection

The analysis of measurement data 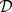 using ODE-MMs requires the estimation of the parameters 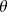. For this we will use maximum likelihood estimation. The likelihood function is the product of the conditional probability of the snapshot data 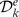 given the parameters 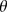. The resulting optimisation problem in terms of the log-likelihood function 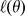 is
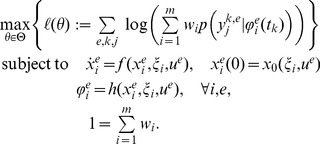
(6)Note that in contrast to the MMs we sum over all combinations of 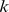 and 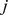, meaning that all time points and experimental conditions are studied simultaneously.

Optimisation problem (6) belongs to the class of ODE constrained optimisation problems. In general this problem is non-convex and possesses local maxima. To determine the parameter vector 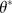 which maximises the log-likelihood function, global optimisation methods are required. Commonly used global optimisation methods are multi-start local optimisation [33], evolutionary and genetic algorithms [34], particle swarm optimisers [35], simulated annealing [36] and hybrid optimisers [37], [38]. For details we refer to available comprehensive surveys of local and global optimisation procedures [33], [39]–[41]. In the following, we will use multi-start local optimisation, an approach which has been shown to be efficient for parameter estimation in RRE models [33].

As the measurement data are limited, the parameters can often not be determined uniquely. In particular the kinetic rates, 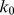 and 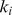, as well as the population fraction, 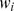 often remain uncertain. A variety of methods exist to assess parameter uncertainties, including profile likelihoods [33], [42], bootstrapping [43], [44], Markov chain Monte Carlo sampling [45], [46], Approximate Bayesian Computing [47], [48] and local approximation to the objective function [44]. In the remainder, we use profile likelihoods due to their often superior efficiency. Profile likelihoods allow for a global uncertainty analysis of individual parameters by means of repeated optimisation. For details we refer to the work of Raue et al. [42].

The source of the cell-to-cell variability, namely the parameters which differ between subpopulations, are often unknown. ODE-MMs can be used to assess the plausibility of different potential sources of cell-to-cell variability by means of model selection. Models corresponding to different hypotheses can be formulated and fitted to the data. The comparison of these models using model selection criteria such as the Akaike information criterion (AIC) [49] or the Bayesian information criterion (BIC) [50] indicates which model is most appropriate. Using such model selection procedures, ODE-MMs can unravel the population structure by predicting differences in properties which have not been measured or are not even measurable. Furthermore, ODE-MMs provide information about rate constants. In contrast, conventional mixture models can only be used to analyse differences in observed quantities.

### Acquisition of snapshot data for NFG-induced Erk1/2 phosphorylation

The proposed ODE-MMs will be used to analyse NGF-induced Erk1/2 phosphorylation. The respective measurement data for NGF-induced Erk1/2 phosphorylation were acquired using quantitative automated microscopy (QuAM) [13]. The preparation of primary sensory neurones from rat (DRG cell culturing), the cell stimulation, the immunofluorescence labelling and the cell imaging was performed according to the protocol described by Andres et al. [19].

In short, primary sensory neurones derived from L1-L6 DRGs were prepared from male Sprague Dawley rats. Dissociated cells were cultured for 15–20 h before stimulated with NGF. After treatment, cells were fixed with paraformaldehyde and permeabilised with Triton X-100. Nonspecific binding sites were blocked and cultures were probed with primary antibodies (anti-phospho-Erk (Thr-202/Tyr-204) (1∶200) and anti-Erk (1∶500)) against target proteins, washed three times, and incubated with secondary antibodies. Cells were quantified with a Zeiss Axioplan 2 microscope controlled by the software Metacyte (Metasystems). As selection marker of sensory neurones, cell identification was performed on immunofluorescently-labelled (Erk staining) cells. The fluorescence intensities derived from pErk antibody and Erk antibody were quantified. To compensate for differences in the mean fluorescence intensity between experimental replicates, the data are normalised.

More detailed information, e.g., information about cell culture conditions as well as the detailed immunofluorescence protocol is provided in Supporting Information S1.

## Results

In the following, we will illustrate how ODE-MMs can be used, how the results can be interpreted and what kind of insights can be gained using them. For this purpose, we study a simulation example for which the ground truth is known as well as an application example for which new biological insights are gained using ODE-MMs.

### Simulation example: Conversion process

**Figure 2:**
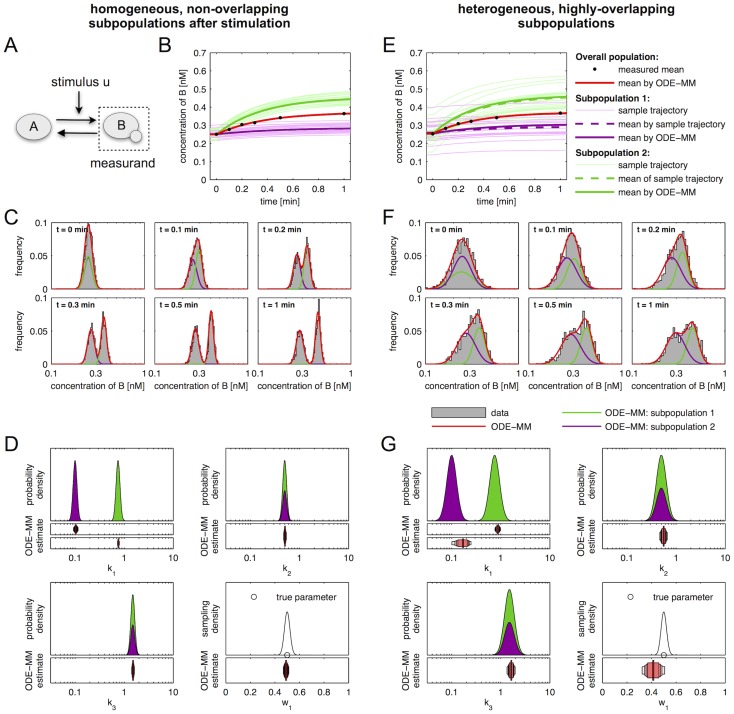
Result of an exemplarily study of a conversion process using ODE constrained mixture modelling. For the conversion process sketched in (A) the cases of homogeneous, non-overlapping subpopulations (B,C,D) and heterogeneous, highly-overlapping subpopulations (E,F,G) are studied. (B,E) Histograms of artificial data for the reversible conversion process (6 time points, 1,000 cells), the best fit achieved using ODE-MM and the distribution predicted for the subpopulations. Artificial data have been generated by sampling single cell parameters from parameter distributions, simulating the single cell model and extracting the concentration of B. ODE-MM was fitted using multi-start local optimisation. (C,F) Representative samples of single cell trajectories for the two subpopulations, the means of the samples and the means for the subpopulations predicted by ODE-MM. (D,G) True parameter distributions (grey shaded area) from which single cell parameters are drawn (purple: subpopulation 1; green: subpopulation 2) and ODE-MM derived parameter estimates including the confidence intervals. Vertical lines mark the maximum likelihood estimates and the horizontal bars represent the confidence intervals corresponding to different confidence levels (80%, 90%, 95% and 99%) computed using profile likelihoods. For the population fraction 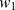, the true value (circle) is shown and the sampling distribution (line) expected for the measured number of cells (1,000), which provides a measure for the expected estimation error.

To illustrate the properties of ODE-MMs and to assess their performance, we consider the conversion process
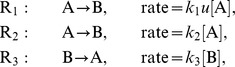
which is illustrated in Figure 2A. The reactions 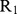 and 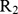 model a stimulus dependent and a stimulus (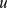) independent (basal) conversion of A to B, respectively. The conversion of 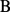 to 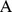 is described by reaction 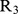. Concentrations of A and B are denoted by [A] and [B]. The conversion of A to B is modulated by the time-dependent concentration 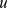 of an external stimulus, also denoted as input. The governing RRE for this conversion process is
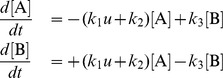
with 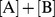 being constant.

#### Population model and artificial data

Artificial data for the conversion process are generated using an ensemble cell population model [51], which belongs to the class of Bayesian hierarchical models. The ensemble consists of single cells whose stimulus response is governed by the RRE stated above. The parameters differ between cells and are drawn from a probability distribution. Ensemble models provide a more detailed description of cell populations and are, in principle, advantageous compared to the ODE-MMs. This description of cell-to-cell variability, namely parameter variability, is thought to be sufficient to describe genetic and epigenetic differences between single cells [51], [52]. However, estimating parameters and parameter distributions of ensemble models is computationally very demanding. Already a single simulation of an ensemble model takes minutes, which limits their application. As ODE-MMs can be simulated orders of magnitudes faster, it is interesting to analyse whether they suffice for extracting the key properties of the underlying subpopulations. To address this question, we consider two scenarios: (1) a cell population consisting of two homogeneous subpopulations which do not overlap after stimulation (Figures 2B–D); (2) a cell population consisting of two heterogeneous, highly-overlapping subpopulations (Figures 2E–G). In both scenarios the subpopulations differ in their responsiveness 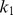 to the stimulation 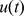. The remaining parameters, 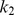 and 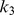, also vary between individual cells but have the same probability distributions across the two subpopulations. The probability distribution of the parameters 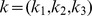 in the individual subpopulations and scenarios is depicted in Figures 2D and G. The initial condition of each cell is the steady state reached for 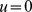 and total concentration, 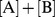, equal to one. At 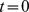, the cells are stimulated, 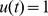 for 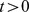, resulting in an increase in the abundance of 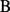. Representative trajectory samples are shown in Figures 2A and D. To obtain the artificial measurement data (Figures 2C and F), the abundance of 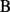 is measured for 1,000 cells at 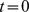, 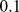, 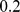, 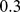, 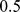 and 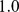.

#### Hypothesis testing

Given the artificial data sets, we first asked whether ODE-MMs can detect the presence of two subpopulations and unravel the differences between them. To address this, we considered four competing hypotheses:

H1 No subpopulations.
H2 Two subpopulations with significantly different stimulus dependent conversion rates 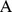 to 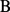 (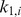 for subpopulation 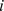).
H3 Two subpopulations with significantly different stimulus independent (basal) conversion rates 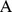 to 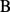 (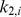 for subpopulation 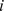).
H4 Two subpopulations with significantly different conversion rates 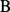 to 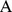 (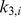 for subpopulation 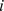).

These four scenarios were described using RRE constrained mixture models. To ensure robustness with respect to the distribution assumption, we considered normal distribution and log-normal distributions with the mean parameterized by the RRE as well as log-normal distributions with the median parameterised by the RRE.

The combination of the 4 hypothesis and the 3 distribution assumptions yields 12 models. These 12 models were fitted to the artificial measurement data using multi-start local optimisation. Components weights were constrained to the interval 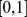, reaction rate constants to 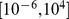, and scale parameters (normal distribution: standard deviation; log-normal distribution: log-standard deviation) to 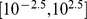. A detailed specification of the models, the maximal value of the log-likelihood function and the BIC values are provided in Table 1. Based on the BIC values, hypotheses H1, H3 and H4 can be rejected. The same holds for the AIC values. The choice of the distribution and its parameterisation plays a role. However, compared to the different model structures, the influence is negligible. Thus, ODE-MMs determined robustly the correct number of subpopulations and even revealed the differences between the subpopulations.

**Table 1:** Parameter estimation and model selection results for conversion process with rather homogeneous subpopulations.

Scenario 1: homogeneous, non-overlapping subpopulations after stimulation										
	*m*	distribution	ODE const.	variability	# par.	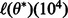	BIC (10^4^)	rank	Δ_BIC_	decision
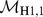	1	normal	mean	-	9	0.9806	−1.9534	10	>10	rejected
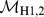	1	log-normal	mean	-	9	0.9785	−1.9493	11	>10	rejected
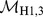	1	log-normal	median	-	9	0.9785	−1.9492	12	>10	rejected
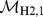	2	normal	mean	*k*_1_	17	1.0998	−2.1848	3	6.215	not rejected
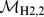	2	log-normal	mean	*k*_1_	17	1.1001	−2.1854	1	0	optimal
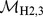	2	log-normal	median	*k*_1_	17	1.1001	−2.1854	2	0.429	not rejected
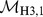	2	normal	mean	*k*_2_	17	0.9911	−1.9673	9	>10	rejected
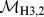	2	log-normal	mean	*k*_2_	17	1.0013	−1.9878	7	>10	rejected
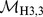	2	log-normal	median	*k*_2_	17	0.9949	−1.9750	8	>10	rejected
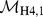	2	normal	mean	*k*_3_	17	1.0087	−2.0026	4	>10	rejected
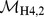	2	log-normal	mean	*k*_3_	17	1.0077	−2.0005	5	>10	rejected
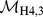	2	log-normal	median	*k*_3_	17	1.0032	−1.9916	6	>10	rejected

For both scenarios (homogeneous subpopulations and heterogeneous subpopulations) four different model hypothesis (H1: no subpopulations; H2: different levels of activatability, 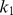; H3: different basal activation rates, 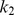; and H4: different deactivation rates, 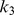) were tested using three models each, differing in the distribution assumption (normal vs. log-normal) and the ODE constrained properties (subpopulation mean vs. subpopulation median). The resulting 12 ODE-MMs were fitted to the experimental data using multi-start local optimisation (accuracy: 10 digits). The plausibility of models has been evaluated using the Bayesian information criterion (BIC) and models were rejected if 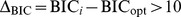 [85]. For both scenarios, ODE-MM unraveled the true underlying population structure (different 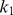 values in the subpopulations) with high significance.

#### Reconstruction of subpopulation characteristics

Following the hypothesis testing, the best models were analysed in greater detail, starting with comparisons of model predictions with the data. This comparison revealed that the measured means (Figures 2B and E) as well as the full distributions (Figures 2C and F) were described well by the selected ODE-MM. Furthermore, although the data did not contain a label to which subpopulation a cell belongs, the ODE-MM derived estimates for mean concentrations of 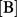 in the subpopulations agreed well with the true means (Figures 2B and E). For scenario 1 (homogeneous subpopulations) the true and the estimated subpopulation means were actually indistinguishable. Hence, ODE-MM derived state estimates for the subpopulation characteristics can indeed be interpreted as average subpopulation characteristics.

#### Interpretation of ODE-MM parameters

Regarding the parameters, we found for scenario 1 that the ODE-MM estimates of the parameters 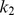 and 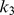 agree with the population average (Figure 2D). For the subpopulation specific parameter 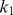, the ODE-MM estimates correspond to the mean parameter in the subpopulation. Even the relative size of the subpopulations is determined well. For scenario 2, in which the subpopulations are more heterogenous and overlap, the inference of the model properties is more challenging and the estimate for the subpopulation size is slightly off (Figure 2G). This can however be explained by the relatively small number of observed cells. As a reference, the distribution of population size observed when sampling 1,000 cells is depicted in Figure 2G. Furthermore, for the subpopulation which responds only weakly to the stimulus, the ODE-MM estimate of the rate constant 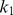 does not correspond to the average rate constant in the subpopulation but overestimates it by a factor of 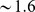. The reason might be a low signal-to-noise ratio. In this subpopulation the basal rate constant 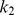 exceeds 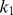 by a factor of 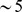. In combination with the large cell-to-cell variability, this might limit the estimation accuracy.

To assess the uncertainty of the parameters, we computed the profile likelihoods. The confidence intervals derived from the profile likelihoods are relatively tight. This indicates that even for cell populations consisting of heterogeneous subpopulations, population snapshots provide information about the dynamical parameters and the subpopulation statistics. Furthermore, for this artificial example, the average parameters in the subpopulation are always within the confidence intervals for the parameters of the ODE-MM. This suggests that the ODE-MM parameters can be interpreted as average parameters of the subpopulations.

To conclude the simulation example, we found that ODE-MMs facilitate the simultaneous analysis of several snapshot data sets. Furthermore, ODE-MMs can be used for hypothesis testing, and the states of the RREs accurately describe the subpopulations while their parameters provide estimates for the means of the underlying biological quantities.

### Application example: NGF-induced Erk1/2 signalling

**Figure 3:**
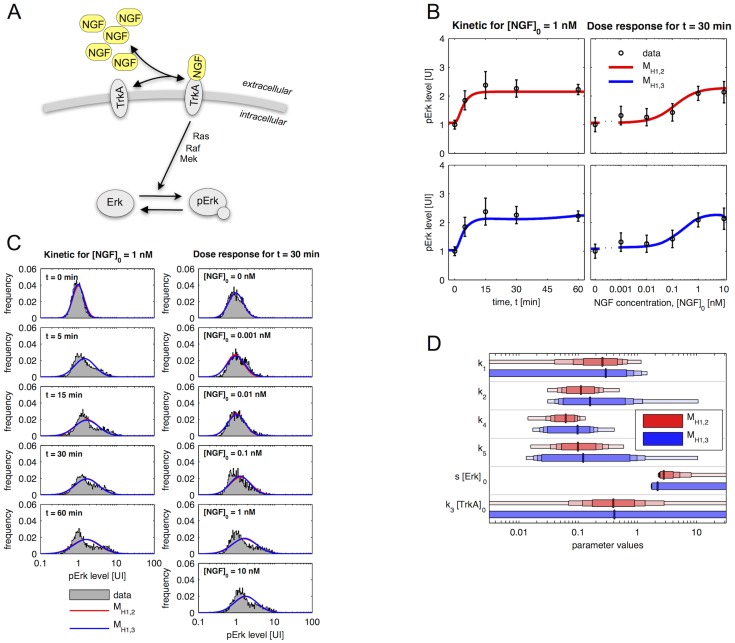
Models for NGF-induced Erk1/2 signalling without subpopulations. (A) Schematic of model for NGF-induced Erk1/2 signalling. Arrows represent conversion reactions and regulatory interactions. (B) Mean and standard deviation of measured pErk levels (kinetic: 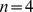, 18797 cells; dose response: 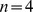, 12205 cells) as well as simulated mean for the models 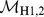 and 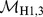. (C) Histograms of the measured pErk levels (data of biological replica are pooled) and corresponding distributions computed using model 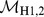 and 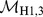. Simulation results for 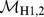 and 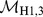 are very similar leading to significant overplotting. pErk levels in (B) and (C) are in arbitrary units of intensity (UI). (D) Maximum likelihood estimates of parameters and confidence intervals for the parameters of 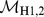 and 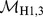. Vertical lines mark the maximum likelihood estimates and the horizontal bars represent the confidence intervals corresponding to different confidence levels (80%, 90%, 95% and 99%) computed using profile likelihoods.

In this section, we use ODE-MMs to perform a data-driven study of NGF-induced Erk1/2 phosphorylation in primary sensory neurones. Primary sensory neurones are commonly used as a cellular model for investigating signalling components mediating pain sensitisation. NGF is known to induce a strong pain sensitisation during inflammation, but also to support neuronal repair during neuropathic pain. Studies showed that NGF binds and activates the receptor tyrosine kinase TrkA [53]. Activation of TrkA leads to the induction of the MAPK/Erk kinase pathway (see Figure 3A) resulting in the phosphorylation of ion channels and protein expression [53].

Beyond the importance of NGF-induced Erk1/2 phosphorylation in pain research, primary sensory neurones are well suited for the evaluation of ODE-MMs as they exhibit a significant degree of cell-to-cell variability. This variability is no nuisance but relevant for their biological function [54]. It has been shown that different neuronal subgroups with different protein abundances and even phosphorylation levels exist [22], [54], namely neurones which detect mechanical stimuli, heat, cold or chemicals. The detailed dynamical characteristics of these subpopulations and the causal differences are largely unknown. In the following, we will employ ODE-MMs to quantify the characteristics of the NGF responsive and unresponsive neuronal subpopulations and their sizes, and to assess reaction rate constants which cannot be obtained experimentally (Figures 3B and C).

#### Experimental data

The quantitative assessment of signalling in primary and heterogeneous cells is challenging compared to cell lines as many experimental methods are not applicable. To study the dynamics of the MAPK/Erk pathway we previously introduced a quantitative automated microscopy technique [13]. This technique allows for the quantification of Erk1/2 activity in single cells and provides rich datasets regarding the cell-to-cell variability. Using this technique we recorded kinetics and dose responses of NGF-induced Erk1/2 phosphorylation [13]. The signals we observed represent relative Erk1/2 phosphorylation, 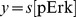, as no calibration curve is employed. The unknown scaling constant which related absolute Erk1/2 phosphorylation, 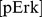, and the measured quantity 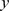 is denoted by 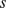.

#### Pathway model

In the literature, it is described that NGF binds to TrkA, yielding the active signalling complex TrkA:NGF. TrkA:NGF-induces the activation of the Ras kinase, which phosphorylates the Raf kinase. The active Raf kinase phosphorylates Mek, which phosphorylates Erk1 and Erk2. In principle the consideration of all these steps is possible, but experimentally the activity of the signalling intermediates Ras, Raf and Mek is difficult to measure in primary sensory neurones as appropriate antibodies are not available. Therefore, we mainly consider a simple pathway model which merely accounts for NGF-TrkA interaction and Erk1/2 phosphorylation. We do not distinguish between Erk1 and Erk2, as their biochemical properties have been demonstrated to be nearly identical (see [55] and references therein). The resulting pathway model A considers five reactions,
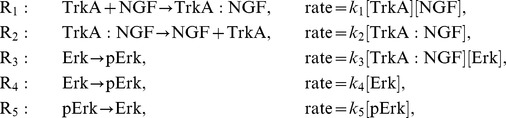
and is illustrated in Figure 3A. The reactions 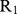 and 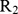 describes binding and unbinding of TrkA and NGF. Basal and TrkA:NGF-induced phosphorylation of Erk are captured by 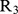 and 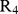. The reactions 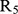 describes the Erk dephosphorylation. By exploiting conservation of mass,
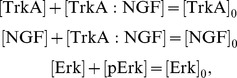
and the well justified assumption that the total NGF concentration is much larger than the total TrkA concentration, 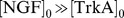, the dynamics of TrkA:NGF and pErk can be stated as
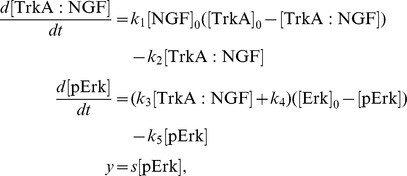
with kinetic parameters 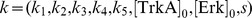. As the absolute concentrations of TrkA and Erk are unknown, this system is structurally non-identifiable. To circumvent this we reformulate the system in terms of 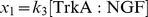 and 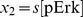, yielding the RRE model
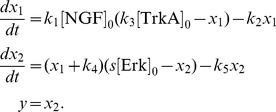
This ODE model merely depends on the products 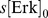 and 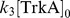 and not on the individual parameters 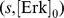 and 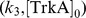, respectively. Thus, we obtain the reduced vector of kinetic parameters 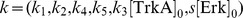.

In the remainder, all plots depict the scaled TrkA:NGF and pErk concentrations, 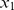 and 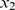.

#### Inference of subpopulation structure

We employed the dynamical pathway model A to assess the population dynamics and to compare the three hypotheses:

H1 No subpopulations.
H2 Two subpopulations with significantly different Erk levels (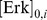 for subpopulation 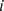).
H3 Two subpopulations with significantly different TrkA levels (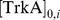 for subpopulation 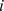).

We only regarded altered abundance of signalling molecules as potential differences between subpopulations. Differences in elementary reaction rates would require mutations or differential post-translational modifications which we consider unlikely. As in the simulation example, the scenarios were described using RRE constrained mixture models. For each scenario we considered normal and log-normal mixture components with means parameterised by the RRE as well as log-normal mixture components with medians parameterised by the RRE. This yielded in total 9 ODE-MMs, which have been fitted using multi-start local optimisation. Properties of models, goodness of fit statistics and obtained BIC values are listed in Table 2. Based upon the BIC (and AIC) values for the different models hypotheses H1 and H2 were rejected compared to H3. Significance levels for the rejections were very high, indicating the presence of two subpopulations with different average levels of TrkA receptors.

**Table 2:** Parameter estimation and model selection results for NGF-induced Erk1/2 signalling (pathway model A).

	*m*	distribution	ODE const.	variability	*# par.*	ℓ 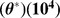	BIC (10^4^)	rank	Δ_BIC_	decision
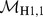	1	normal	mean	-	17	−5.2890	10.5955	9	>10	rejected
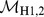	1	log-normal	mean	-	17	−3.7659	7.5495	6	>10	rejected
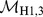	1	log-normal	median	-	17	−3.7556	7.5288	5	>10	rejected
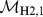	2	normal	mean	[Erk]_0_	30	−4.0348	8.1006	8	>10	rejected
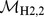	2	log-normal	mean	[Erk]_0_	30	−3.6482	7.3274	4	>10	rejected
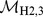	2	log-normal	median	[Erk]_0_	30	−3.6262	7.2835	3	>10	rejected
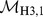	2	normal	mean	[TrkA]_0_	30	−3.9846	8.0002	7	>10	rejected
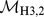	2	log-normal	mean	[TrkA]_0_	30	−3.5847	7.2003	2	2.189	not rejected
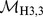	2	log-normal	median	[TrkA]_0_	30	−3.5846	7.2001	1	0	optimal

For each biological hypothesis (H1: no subpopulations; H2: different levels of total Erk, 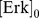; H3: different levels of total TrkA, 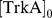) three models, differing in the distribution assumption (normal vs. log-normal) and the ODE constrained properties (subpopulation mean vs. subpopulation median), have been specified and fitted to the experimental data using multi-start local optimisation (accuracy: 10 digits). For the model selection using the Bayesian information criterion (BIC) we found that H1 and H2 could be rejected according to [85] as 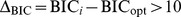. The subpopulations seemed to follow a log-normal distribution. The two models, 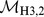 and 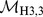, which only differ in the ODE constrained property were acceptable.

We note that the rejection of hypotheses H1 and H2 requires information about the distribution of pErk levels. Even models for the simplest hypothesis, H1, describe the kinetic and dose response of the mean pErk level (Figure 3B). This proves that the mean is not informative enough and implies that simple RRE models are in general insufficient for determining subgroups. Using the distribution of pErk levels in combination with ODE-MMs, H1 can be rejected easily (Figure 3C) due to the strong model-data mismatch for stimulation with 1 nM and 10 nM NGF.

#### Size and characteristics of subpopulations

**Figure 4:**
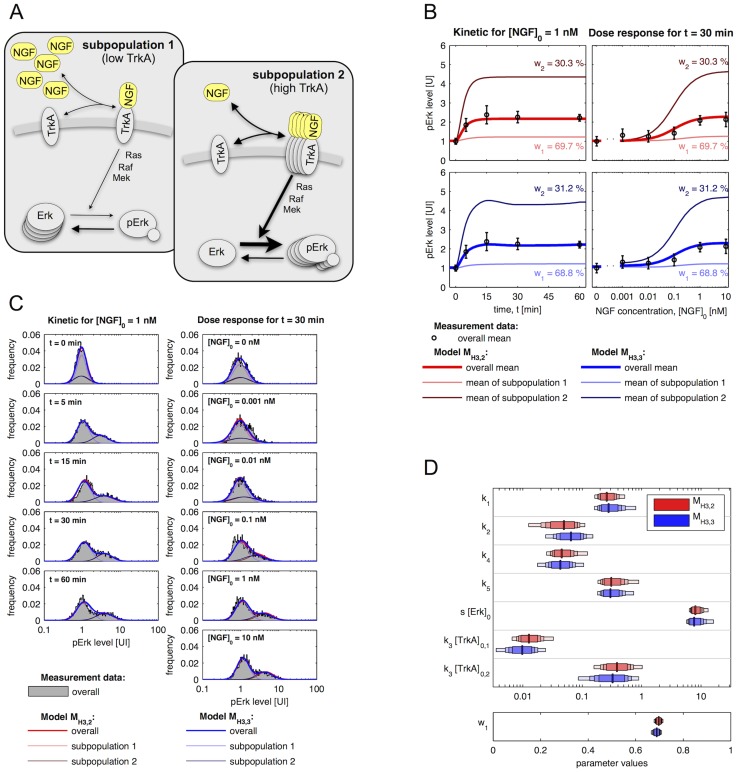
Models for NGF-induced Erk1/2 signalling with two subpopulations which have different TrkA levels. (A) Schematic of model for NGF-induced Erk1/2 signalling. Arrows represent conversion reactions and regulatory interactions. The frequency of an object is used to illustrate its abundance. (B) Mean and standard deviation of measured pErk levels (kinetic: 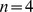, 18797 cells; dose response: 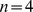, 12205 cells) as well as simulated mean for the models 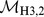 and 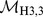. (C) Histograms of the measured pErk levels (data of biological replica are pooled) and corresponding distributions computed using model 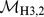 and 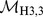. Simulation results for 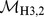 and 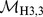 are very similar leading to significant overplotting. pErk levels in (B) and (C) are in arbitrary units of intensity (UI). (D) Maximum likelihood estimates of parameters and confidence intervals for the parameters of 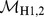 and 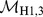. Vertical lines mark the maximum likelihood estimates and the horizontal bars represent the confidence intervals corresponding to different confidence levels (80%, 90%, 95% and 99%) computed using profile likelihoods.

The selected population structure, H3, assumes different concentrations of the NGF receptor TrkA for the subpopulations. This results in different concentration of TrkA-NGF complexes and ultimately in different Erk phosphorylation levels. The overall Erk concentration, [Erk] + [pErk], is the same for the subpopulations. An illustration of the models and signalling is provided in Figure 4A.

The ODE-MMs representing H3 explain the kinetic and dose response measurements of the mean pErk concentration as well as the pErk distribution. Measurement data and fits for the best two models, 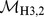 and 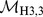, are depicted in Figures 4B and C. These two models exploit different parameterisations of the log-normal distributions (see Table 2). In 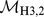 the mean is parameterised using the RRE, while in 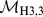 the median is parameterised. The latter yields a slightly better fit, however, the differences are minor and not statistically significant. As 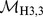 does not describe the time-dependent mean, splines are used to obtain Figure 4B (bottom).

The maximum likelihood estimation of the model parameters provides estimates for the relative size of the subpopulations and their pErk levels. Roughly 70% of the cells belong to the subpopulation with low TrkA levels (subpopulation 1) and 30% of the cells possess high TrkA levels (subpopulation 2). Subpopulation 1 hardly responds to NGF, while subpopulation 2 responds with a 4-fold increase in pERK levels for a 1 nM NGF stimulation. The maximal response is reached after 10 minutes and the response amplitude saturates for NGF concentration 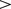
1 nM. The differences between the subpopulations sound large, however, a direct extraction of these insights from the data is impossible due the the large overlap of subpopulations. This renders the proposed ODE-MMs, which incorporate pathway information, essential.

#### Quantification of kinetic parameters and abundance differences

Beyond subpopulation differences in observed pErk levels, ODE-MMs rendered quantities accessible which could not be measured. In particular the Erk dephosphorylation rate and the NGF-TrkA affinities could be inferred. Furthermore, we found a 30-fold difference between TrkA levels in the two subpopulations. This information is valuable as TrkA antibodies with high sensitivity and specificity are not available for immunofluorescence based experiments in cultures of primary sensory neurones. A practical identifiability analysis using profile likelihood showed that all estimated parameters – kinetic parameters, subpopulation sizes and standard deviations – are identifiable (Figure 4D; and Figures 1 and 2 in Supporting Information S1). Indeed, the confidence intervals for most parameters, in particular the subpopulation sizes and standard deviations, are rather narrow (Tables 2, 3 and 4 in Supporting Information S1). This and the rather consistent estimates obtained using different models (Supporting Information S1), indicate the reliability of the parameter estimates.

The ODE-MMs 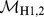 and 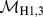 for H1 possess 17 parameters while the ODE-MMs 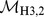 and 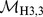 for H3 possess 30 parameters. As the ODE-MMs for H1 possess 13 parameters less than the ODE-MMs for H3 we expected that the parameters of 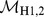 and 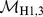 are more well determined than the parameters of 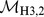 and 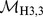. The comparison of parameter uncertainties for H1 (Figure 3D) and for H3 (Figure 4D) yielded however a surprising, counterintuitive result. While the kinetic parameters of 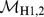 and 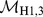 were mostly practically non-identifiable, all parameters of 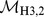 and 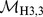 were practically identifiable. A possible explanation is that ODE-MMs for H1 cannot exploit all information encoded in the distribution as the model is not flexible enough, rendering the data less informative and causing non-identifiability of parameters. This on the other hand means that the informativeness of data depends on the model used to analyse them. More flexible models might not only provide deeper insights but also provide more reliable estimates.

#### Comparison of pathway models

**Figure 5:**
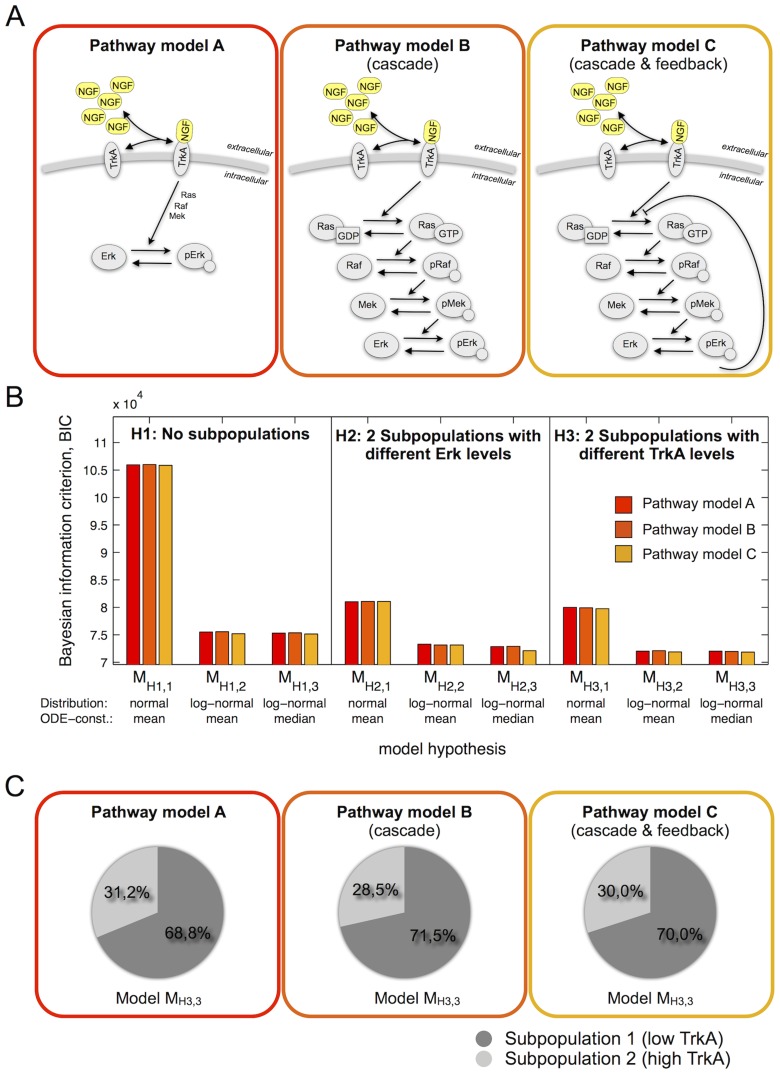
Comparison of three different pathway models for the NGF-induced Erk1/2 activation. (A) Schematics of three model for NGF-induced Erk1/2 activation. Pathway model A is a simple two component model, while pathway models B and C contain a detailed description of the signalling cascade. Pathway model C also accounts for a negative feedback from pErk to Ras activation. (B) Comparison of different pathway models (colour-coded), hypotheses about the cell-to-cell variability (H1, H2 and H3) and distribution assumptions (distribution: normal vs. log-normal; ODE-constrained: mean or media). BIC values indicate that differences between the pathway models are small compared to differences arising from different variability hypotheses and distribution assumptions. (C) Maximum likelihood estimates of the subpopulations sizes found for each pathway model.

Pathway model A (Figure 5A, left) which we studied so far merely accounts for TrkA and Erk dynamics. In the literature more detailed models for NGF-induced Erk1/2 activation have been proposed [56]–[60]. Although these pathway models have been developed for cell lines, such as the rat pheochromocytoma cell line (PC12), we expect the structure of the pathway to be similar in primary DRG neurones. In contrast, protein abundances and reaction rates are most likely altered, which limits the reusability of the available quantitative information. In addition, cell lines are most likely more homogeneous than the primary DRG neurons considered in this project.

To evaluate the robustness of our predictions with respect to the choice of the pathway description, we considered two additional pathway models. Pathway models B and C (Figure 5A, middle and right) account for the signal amplification cascade and a negative feedback, two key features of the NGF-induced Erk1/2 activation pathway. These models are therefore more flexible and possess a larger number of unknown parameters. For details on the models and their mathematical descriptions we refer to the Supporting Information S1.

As for pathway model A, we carried out the parameter estimation and model selection for pathway models B and C (Supporting Information S1). Interestingly, for all pathway models we found the same ranking of subpopulation structures. Two subpopulations with different TrkA concentrations, log-normally distributed pErk levels and ODE-constrained subpopulation median (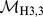) were always preferred. Furthermore, our comparison of BIC values (Figure 5B) revealed that the influence of the pathway model on the BIC values is small in comparison with the influence of the subpopulation structure (H1–H3) and the distribution assumptions. This indicates that for an accurate description of the measurement data, the subpopulation structures is more important than a more detailed description of the signalling pathway.

The hypothesis testing using different pathway models supported our prediction that TrkA is the key source of cell-to-cell variability. Moreover, the maximum likelihood estimates for the size of the responsive subpopulations (Figure 5C) were consistent. For the kinetic parameters such a comparison was not possible as (i) the meaning of parameters differ between pathway models and (ii) many parameters of the pathway models B and C are non-identifiable. As for more detailed pathway models we expect that the parameter identifiability becomes even worse, we did not study the most detailed and sophisticated model for the NGF signalling pathway [58], [59].

#### Validation of subpopulation structure

To validate the ODE-MM derived prediction that subpopulations do not possess different Erk levels (H2) but different TrkA levels (H3), co-labelling experiments have been performed. In addition to Erk phosphorylation also total Erk is quantified using a second antibody. As both measurements provide only relative information the scales are not comparable. For details regarding the experiments, we refer to the section *Materials and Methods* in Supporting Information S1.

**Figure 6:**
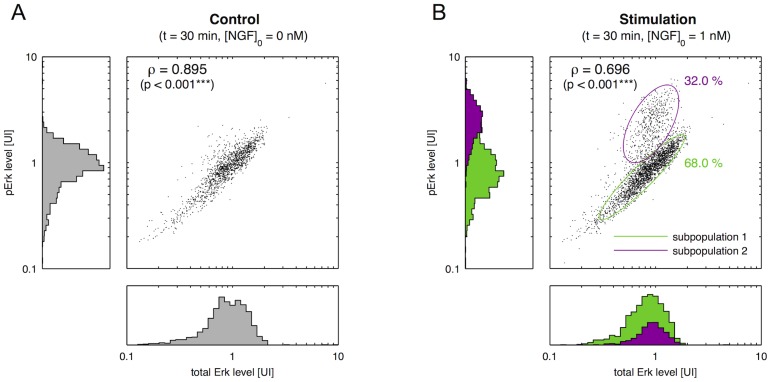
Two-dimensional analysis of Erk and pErk levels. Joint distribution of pErk levels and total Erk levels under (A) control conditions and after (B) stimulation with 1 nM NFG for 30 minutes, along with the corresponding histograms (pooled data of 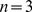 biological replicates, 4134 cells). Measured pErk levels, 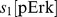, and total Erk levels, 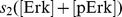, are in arbitrary units of intensity. (B) Data measured after stimulation with 1 nM NGF have been fitted with a 2-component normal mixture model, of which the level set and the components weights are depicted. Using the mixture model the measured cells are assigned to the subpopulations and the corresponding contribution to the histogram are colour-coded.

Figures 6A and B depict the distribution of pErk and total Erk levels observed under control conditions and after stimulation with 1 nM NGF for 30 minutes. As expected, cells with high total Erk levels tend to possess high pErk levels. The Pearson correlation is 0.895 for the control and 0.696 for the stimulation. The significant correlation decreases after NGF stimulation is caused by the appearance of a group of cells with high pErk signals. To analyse the NGF-induced response in more detail we fit a simple 2-dimensional mixture of normal distribution to the logarithmized data. Figure 6B shows the level sets of the two mixture components, which are denoted by subpopulations 1 and 2. By comparing Figures 6A and B we found that subpopulation 1 is similar to the control population. Hence, subpopulation 1 hardly responds to the NGF stimulation. In contrast, subpopulation 2 has a significantly increased average pErk level.

As subpopulations 1 and 2 have similar total Erk but different pErk distributions, total Erk is not the cause of the different activation potentials of the subpopulations. This verifies the rejection of hypothesis H2, which assumed a predominant role of the total Erk. The different activation potentials have to be caused by a further network compound such as TrkA. This partially validates hypothesis H3. However, the differences could in principle also be due to intermediate signalling components, such as Raf and Mek, which are not considered in the model. While a conclusive proof of H3 would require a simultaneous labelling of pErk and TrkA, which is currently infeasible due to the lack of appropriate TrkA antibodies, there are three – in our opinion convincing – indications that TrkA causes the population split. First of all, the available measurement data can be described by assuming different TrkA levels. Secondly, the estimate for the fraction of cells with high TrkA levels (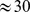
%) derived via ODE-MMs from the pErk kinetic and dose response (Figure 4B) agrees perfectly with the size of the responsive subpopulation found by co-labelling pErk and Erk (subpopulation 2, Figure 6B). The size of the responsive subpopulation has been determined from the co-labelling data using mixture modelling, which yields for this 2D data robust results as the subpopulations are rather different. Finally, Kashiba et al. [61] found that 35% of primary sensory neurones are TrkA positive, which is in good agreement with the size of the responsive subpopulation we found using ODE-MM and co-labelling.

To conclude, in this section we proved the applicability of ODE-MMs to practically relevant biological problems. We used ODE-MMs to study data from primary sensory neurones and to determine subpopulation characteristics and kinetic rates. Furthermore, we provided a data-driven explanation for the observed cell-to-cell variability and validated this explanation partially using new experimental data.

## Discussion

Most multicellular organisms and microbial colonies consist of subpopulations with distinct biological functions. A study of mechanistic differences between these subpopulations and their functions is crucial for a holistic understanding of such complex biological systems. In this work, we introduced ODE constrained mixture models, a novel class of data analysis tools which can help to detect subpopulations and to analyse differences between them using population snapshot data. A simulation example illustrates that ODE-MMs possess a higher sensitivity than classical mixture models and ODE models, which originates from the simultaneous exploitation of distribution information and dependencies between experimental conditions. Furthermore, ODE-MMs provide mechanistic insights, e.g., estimates for kinetic parameters and abundance differences between subpopulations. In contrast to population models relying on a stochastic description of the individual cell [62]–[64] or ensemble models with parameter distributions [65], [66], which can in principle also be used to analyse systems with different subpopulations, the computation time is significantly reduced. Furthermore, ODE-MMs are easily applicable as they merely rely on ODE models, for which numerical simulation as well as parameter estimation is well established [33].

To assess and illustrate the properties of ODE-MMs, we studied the response of primary sensory neurones to NGF stimulation. Therefore, we considered single-cell data for Erk1/2 phosphorylation levels collected by quantitative automated microscopy (QuAM) [13], [18]. Using these data we performed model selection and found that the cell population consists of two subpopulations with different abundances of the NGF receptor TrkA. The responsive subpopulation with high TrkA levels constituted 30% of the overall population. By performing co-labelling experiments in which pErk1/2 and total Erk1/2 have been measured, we validated the existence of two subpopulations and found strong indications that TrkA is the causal factor for the population split. Thus, ODE-MMs enabled the inference of the population structure using only measurement of pErk1/2. Even the estimated size of the subpopulation with high TrkA expression was consistent with the newly collected as well as the literature data. This implies that ODE-MMs have the potential to significantly reduce the number of different measurements required to analyse heterogeneous populations and are even capable of predicting causal factors for the population split which have not been observed.

Beyond insights in subpopulation substructures, ODE-MM can improve estimates of kinetic parameters. This has been revealed by a profile likelihoods based uncertainty analysis of ODE-MMs for NGF-induced Erk1/2 phosphorylation. We found that kinetic parameters of ODE-MMs with two subpopulations are better identifiable than kinetic parameters of ODE-MMs without subpopulation structure. In many situations additional model complexity and an increased number of parameters results in increased parameter uncertainty. This is however not the case if the more complex model can exploit additional features of the data. In this case the data are effectively more informative for a more complex model resulting in a reduced parameter uncertainty. We are not aware of papers which reported this generic observation.

For our analysis of NGF-induced Erk1/2 phosphorylation we considered three pathway models. While these models consider key network motifs, such as an amplification cascade and a negative feedback loop, they are simple compared to the most detailed models (see [56]–[60] and references therein). These more detailed models have however been developed for cell lines and it is unclear how well they describe the signalling in primary sensory neurones. Furthermore, all three models we studied fit the experimental data and provided consistent predictions for the population structure, indicating a certain degree of robustness with respect to the pathway model. However, model extension may become necessary if the amount of available measurement data for primary sensory neurones increases, other stimuli are included or the biological question changes.

In this study we employed reaction rate equation models to constrained means and medians of mixture components. A further improvement of the sensitivity of ODE-MMs might be achieved by using ODE models which capture the cell-to-cell variability within subpopulations. Possible choices are linear noise approximations [27], [28], effective mesoscopic rate equations [29], [30] or moment equations [31], [32]. These ODE models allow for an improved mechanistic description of the single cell dynamics, in particular the explicit consideration of intrinsic and/or extrinsic noise [67]. Intrinsic noise is related to the stochasticity of biochemical reactions. Extrinsic noise can originate from variation outside the considered signalling pathway and can be related to cell size, cell cycle state or the history of a cell. A variety of modelling approaches has been proposed for systems exhibiting intrinsic noise [1], [62]–[64], [68]–[70], extrinsic noise [51], [65], [71]–[73] and combinations of both [52], [74], [75]. The aforementioned deterministic, ODE-based approximation of these modelling approaches could build the basis for the description of the subpopulation dynamics. The consideration of more general ODE constraints describing the temporal correlation of stochastic processes [76], [77] might even allow for the study of single-cell dynamics based on time-lapse microscopy data. In this context explicit models of the measurement noise might be beneficial, which have not been considered here, as the covariance was nevertheless a free parameter.

Consistent with our studied biological applications, we considered the special case of constant population sizes. There are however many situations in which spontaneous [5] or stimulus-induced cell-type transitions [5], [10], [78] occur. While such scenarios have not been considered in this manuscript and are not captured by our formulation, ODE-MMs can be generalised to studying such cell systems. Changing subpopulation sizes might be captured using parametric functions, splines or dynamic mechanistic models.

In our studies, ODE-MM parameters have been estimated by solving the maximum likelihood problem using multi-start local optimisation. The computational efficiency of this approach could probably be improved by using expectation maximisation (EM) algorithms [79]. Also the profile likelihood-based uncertainty analysis approach we used would profit from this. To obtain uncertainty bounds not only for parameters but also for model predictions, prediction profile likelihoods [80] or Bayesian methods [46] can be used.

The availability of pathway information in databases like KEGG [81], BioPath [82], BioCyc [83] and others is steadily increasing. We illustrated that integrating this information with snapshot data yields additional insights. ODE-MMs are however not only applicable to pure snapshot datasets but can be used to analyse mixed sets of snapshot and population average data (e.g., Western blots). Furthermore, we expect that the methods scale well. Solely, the numerical simulation of the ODE models is critical, but for this, efficient and reliable solvers exist which can easily handle systems with hundreds of chemical species [84]. Therefore, ODE-MMs should be applicable to large-scale datasets, such as transcriptomics, proteomics and metabolomics. This renders ODE-MMs potentially very valuable for the analysis of heterogeneous groups, not only cell populations, but also patient cohorts.

## Supporting Information

Code S1 **MATLAB code used for ODE constrained mixture modelling.** This zip-file contains the MATLAB code for the simulation example (conversion process) and the application example (NGF-induced Erk1/2 phosphorylation) presented in the paper. We provide implementations for the models, the parameter estimation, the uncertainty analysis and the model selection. In addition to the implementation, also all data and result files (.mat) are included.
(ZIP)

Supporting Information S1 **Supplemental notes regarding the computational modelling.** This document provides a detailed description of the different pathway models, the parameter estimation, the uncertainty analysis and the model selection. Furthermore, numerical results of the parameter estimation, the uncertainty analysis and the model selection are listed and illustrated.
(PDF)
