# Supplementary material for: ODE Constrained Mixture Modelling: A Method for Unraveling Subpopulation Structures and Dynamics
Source: PLoS Comput Biol. 2014 Jul 3;10(7):e1003686. doi: 10.1371/journal.pcbi.1003686 (PMC4081021; doi:10.1371/journal.pcbi.1003686)
Supplement: Supporting Information S1 — Supplemental notes regarding the computational modelling. This document provides a detailed description of the different pathway models, the parameter estimation, the uncertainty analysis and the model selection. Furthermore, numerical results of the parameter estimation, the uncertainty analysis and the model selection are listed and illustrated. (PDF) [file pcbi.1003686.s002.pdf]

Supplement:

ODE constrained mixture modelling: A method for unraveling subpopulation structures and dynamics

Jan Hasenauer[1,2,*], Christine Hasenauer[3], Tim Hucho[3,4], and Fabian J. Theis[1,2]

[1]Institute of Computational Biology, Helmholtz Center Munich, Munich, Germany

[2]Division of Mathematical Modeling of Biological Systems, Department of Mathematics, University of Technology Munich, Munich, Germany

[3]Max Planck Institute for Molecular Genetics, Berlin, Germany

[4]Division of Experimental Anesthesiology and Pain Research, Department of Anesthesiology and Intensive Care Medicine, University Hospital Cologne, Cologne, Germany

[*]corresponding author (e-mail: jan.hasenauer@helmholtz-muenchen.de)

Contents

1 Material and Methods **2**
- 1.1 Chemicals . . . 2
- 1.2 Antibodies . . . 2
- 1.3 Animals . . . 2
- 1.4 DRG-cultures . . . 2
- 1.5 Cell stimulation . . . 2
- 1.6 Immunocytochemistry . . . 2
- 1.7 Quantitative Automated Microscopy (QuAM) . . . 3
- 1.8 Data preprocessing . . . 3

2 Parameter estimation, uncertainty analysis and model selection **3**

3 Models for the NGF signalling pathway **3**
- 3.1 Pathway model A . . . 4
  - 3.1.1 Reaction network . . . 4
  - 3.1.2 Reaction rate equation and parametrization . . . 4
  - 3.1.3 Parameter estimation . . . 4
- 3.2 Pathway model B . . . 5
  - 3.2.1 Reaction network . . . 5
  - 3.2.2 Reaction rate equation and parametrization . . . 5
  - 3.2.3 Parameter estimation . . . 6
- 3.3 Pathway model C . . . 7
  - 3.3.1 Reaction network . . . 7
  - 3.3.2 Reaction rate equation and parametrization . . . 7
  - 3.3.3 Parameter estimation . . . 8

4 Implementation **8**

1 Material and Methods

1.1 Chemicals

BSA, L-glutamine, poly L-ornithine hydrochloride, DMSO, paraformaldehyde, Triton X-100 and glutamate were purchased from Sigma (Taufkirchen, Germany), collagenase P from Roche (Mannheim, Germany), trypsin from Worthington Biochemical Corporation (Freehold, NJ, USA), Neurobasal A (without phenol red), B27 supplement, laminin, minimum essential medium with glutamax were purchased from Invitrogen (Germany, UK) or Life Technologies (Grand Island, NY, USA), DMEM, trypsin and EDTA from Clonetics (Cambrex, US) and normal donkey serum from Dianova (Hamburg, Germany). mNGF was purchased from Alomone (Jerusalem, Israel).

1.2 Antibodies

Anti-phospho-Erk (Thr-202/Tyr-204) was purchased from New England Biolabs (Frankfurt am Main, Germany; final concentration 1:200). Anti-Erk was purchased from BD Bioscience (Heidelberg, Germany; final concentration 1:500). Alexa-594-labelled chicken anti-rabbit IgG and 633 goat anti-mouse IgG were purchased from Molecular Probes / Invitrogen (Karlsruhe, Germany; final concentration 1:1000 and 1:500, respectively).

1.3 Animals

Male Sprague Dawley rats were purchased from Harlan (220-240 g; Rossdorf, Germany). Our institution is licensed to house and work with these animals by the responsible authority (LaGeSo, Berlin, license ZH120). For tissue collection, rats were euthanized by CO_2 inhalation. This procedure was reported to and approved by the LAGeSo, Berlin (T0370/05). All efforts were made to minimise the number of animals used and their suffering.

1.4 DRG-cultures

Dissociated DRG neurones were prepared from male Sprague Dawley rats. Rats were killed by CO_2 intoxication and L1-L6 DRGs were removed, desheathed, pooled, and incubated with collagenase (final concentration (f.c.) 0.125%; 1 h, 37 °C). The neurones were dissociated by trypsin digestion (f.c. 0.25%, 8 min, 37 °C) and trituration with a fire-polished Pasteur pipette. Axon stumps and dead cells were removed by centrifugation (5 min, 100 g). Viable cells were resuspended in 12 ml of NeurobasalA/B27 medium and plated 0.5 ml/culture onto polyornithine/laminin-precoated glass coverslips (12 mm diameter) and incubated overnight in 24 well plates at 37 °C in 5% CO_2.

1.5 Cell stimulation

Cells were cultured for 15-20 h before stimulated with NGF. To ensure homogeneous mixing of the stimulant with the cell culture medium, half the media in each well (250 μl) was removed, mixed thoroughly with the stimulant, and added back to the same well. Negative controls were treated alike but without the addition of any reagent. To reduce mechanical cell stress the medium was added back to the cultures very slowly (250 μl in 6 s) using an automatic pipette (Multipette pro, Eppendorf). After treatment, cells were washed once with phosphate-buffered saline (PBS) and fixed with paraformaldehyde (4%, 10 min) at room temperature (RT).

1.6 Immunocytochemistry

Paraformaldehyde-fixed cells were permeabilised with 0.1% Triton X-100 (10 min, RT), followed by three washes with PBS (5 min, RT). After blockage of nonspecific binding sites (5% bovine serum albumin (BSA) and 10% normal donkey serum in PBS; 1 h, RT), the cultures were probed with primary antibodies against target proteins (antibody concentrations as indicated in Methods, Antibodies section) in 1% BSA in PBS (1 h, RT), washed three times (1% BSA in PBS; 5 min, RT), and incubated with secondary antibodies (1 h, RT). After three final washes (PBS; 5 min, RT), the cultures were mounted with Fluoromount-G (Southern Biotech / Biozol) containing DAPI (0.5 μg/ml).

1.7 Quantitative Automated Microscopy (QuAM)

As published recently, cells were evaluated with a Zeiss Axioplan 2 microscope controlled by the software Metacyte (Metasystems) [1]. In short, images of mounted coverslips were taken (1280 × 1024 pixels) with a 10× objective. The exposure time was defined automatically so that maximal 1000 pixel / 100 μm^2 were saturated, but with an exposure time of maximal 0.96 s. As selection marker of sensory neurones, cell identification was performed on immunofluorescently-labeled (Erk staining) cells. Parameters were defined to automatically identify neurones but not glia cells, (positive selection parameters: size 150-1500 μm^2, form (aspect ratio = 2; concavity depth = 0.25) and contrast (object threshold 30%). The pixel intensity of selected neurones was averaged, and normalised against object area and exposure time. The fluorescence intensities derived from pErk antibody and Erk antibody were quantified.

1.8 Data preprocessing

Experimental and biological variability causes differences in the mean fluorescence intensity between experimental replicates. To compensate for these differences, the data are normalised. All fluorescence values collected on one day are scaled with a constant, which is chosen such that quadratic mismatch of the fluorescence mean intensities across replicates is minimal. For details we refer to the implementation (`data_scaling.m`).

Most research groups determine the scale constant using only the control. We however observed, that more robust results are achieved when considering all data for the normalisation.

2 Parameter estimation, uncertainty analysis and model selection

The parameter estimation for the ODE-MM models is performed using multi-start local optimisation. At least 100 runs of the local optimiser were performed for each model. If the best objective function value has not been determined at least 5 times, the number of local optimisations has been increased. For computational efficiency, all parameters besides the subpopulation weights have been log-transformed. The bounds for the optimisation variables are provided in the next section.

For the analysis of parameter uncertainties we used *asymptotic confidence intervals* as well as *finite sample confidence intervals*. Asymptotic confidence intervals were computed from the curvature of the likelihood at the maximum likelihood, namely the Hessian. Finite sample confidence intervals were determined using profile likelihoods (see [2] for details).

The model selection has been performed using the Bayesian information criterion (BIC) [3]. The results of the model selection are summarised in Supplement Table 1.

3 Models for the NGF signalling pathway

In the following we introduced three models for the NGF signalling pathway. These models are rather simple and rely on a minimal set of assumptions and parameters.

In the literature, detailed models for the NGF-induced Erk1/2 phosphorylation have been proposed. These available models have been developed to study the response of the rat pheochromocytoma cell line (PC12) to NGF stimulation. In this study we considered however primary sensory neurones derived from rat DRGs. It has been shown that the response characteristics of PC12 cells and primary DRG neurones differ [1].

We believe that the pathway topology is similar in PC12 cells and primary DRG neurones, protein abundances and reaction rates are however most likely altered. This can shift the pathway in a completely different dynamic regime, in which other mechanism are important, and limits the reusability of the available quantitative information. Besides the limited amount of experimental data for primary sensory neurones, the limited reusability of existing information is the key reason why we start out with rather simple models.

3.1 Pathway model A

The pathway model A provides a simple description of NGF-induced Erk1/2 phosphorylation which merely accounts for the NGF receptor TrkA and Erk.

3.1.1 Reaction network

Pathway model A accounts for the reactions,

$$\begin{aligned}
\mathrm{R}_1: &\quad \text{TrkA} + \text{NGF} \to \text{TrkA:NGF}, && \text{rate} = k_1[\text{TrkA}][\text{NGF}],\\
\mathrm{R}_2: &\quad \text{TrkA:NGF} \to \text{NGF} + \text{TrkA}, && \text{rate} = k_2[\text{TrkA:NGF}],\\
\mathrm{R}_3: &\quad \text{Erk} \to \text{pErk}, && \text{rate} = k_3[\text{TrkA:NGF}][\text{Erk}],\\
\mathrm{R}_4: &\quad \text{Erk} \to \text{pErk}, && \text{rate} = k_4[\text{Erk}],\\
\mathrm{R}_5: &\quad \text{pErk} \to \text{Erk}, && \text{rate} = k_5[\text{pErk}],
\end{aligned}$$

and the mass conservation

$$\begin{aligned}
[\text{TrkA}] + [\text{TrkA:NGF}] &= [\text{TrkA}]_0\\
[\text{NGF}] + [\text{TrkA:NGF}] &= [\text{NGF}]_0\\
[\text{Erk}] + [\text{pErk}] &= [\text{Erk}]_0.
\end{aligned}$$

3.1.2 Reaction rate equation and parametrization

Under the assumption that the total NGF concentration is much larger than the total TrkA concentration, $[\text{NGF}]_0 \gg [\text{TrkA}]_0$, the dynamics of TrkA:NGF and pErk can be stated as

$$\begin{aligned}
\frac{d[\text{TrkA:NGF}]}{dt} &= k_1[\text{NGF}]_0([\text{TrkA}]_0 - [\text{TrkA:NGF}]) - k_2[\text{TrkA:NGF}]\\
\frac{d[\text{pErk}]}{dt} &= (k_3[\text{TrkA:NGF}] + k_4)([\text{Erk}]_0 - [\text{pErk}]) - k_5[\text{pErk}]\\
y &= s[\text{pErk}],
\end{aligned}$$

with kinetic parameters $k = (k_1, k_2, k_3, k_4, k_5, [\text{TrkA}]_0, [\text{Erk}]_0, s)$. As the absolute concentrations of TrkA and Erk are unknown, this system is structurally non-identifiable. To circumvent this we reformulate the system in terms of $x_1 = [\text{TrkA:NGF}]/k_3$ and $x_2 = s[\text{pErk}]$, yielding the ODE model

$$\begin{aligned}
\frac{dx_1}{dt} &= k_1[\text{NGF}]_0(k_3[\text{TrkA}]_0 - x_1) - k_2x_1\\
\frac{dx_2}{dt} &= (x_1 + k_4)(s[\text{Erk}]_0 - x_2) - k_5x_2\\
y &= x_2.
\end{aligned}$$

This ODE model merely depends on the products $s[\text{Erk}]_0$ and $k_3[\text{TrkA}]_0$ and not on the individual parameters $(s, [\text{Erk}]_0)$ and $(k_3, [\text{TrkA}]_0)$, respectively. Thus, we obtain the reduced vector of kinetic parameters $k = (k_1, k_2, k_4, k_5, k_3[\text{TrkA}]_0, s[\text{Erk}]_0)$.

As initial condition we use the steady state of this system for $[\text{NGF}]_0 = 0$,

$$\begin{aligned}
x_1(0) &= 0\\
x_2(0) &= \frac{k_4(s[\text{Erk}]_0)}{k_4 + k_5}.
\end{aligned}$$

3.1.3 Parameter estimation

For the parameter estimation and model selection we constrained the kinetic parameter k_i to the interval $[10^{-10}, 10^{10}]$. The subpopulation sizes w_i have been constrained to $[0, 1]$. Standard deviations of normal distributions and shape parameters of log-normal distributions were constrained to $[10^{-1}, 10^2]$ and $[10^{-1}, 10^1]$, respectively.

Maximum likelihood estimates and profile likelihood derived confidence intervals are provided in Table 2-4. For the best two models, $\mathcal{M}_{\text{H3},2}$ and $\mathcal{M}_{\text{H3},3}$, profile likelihoods and their local approximations are depicted in Supplement Figures 1 and 2.

3.2 Pathway model B

The pathway model B is more detailed than pathway model A and accounts for the protein amplification cascade from TrkA to Erk via Ras, Raf and Mek.

3.2.1 Reaction network

Model B accounts for the reactions,

$$
\begin{aligned}
&R_1: && \text{TrkA} + \text{NGF} \to \text{TrkA:NGF}, && \text{rate} = k_1[\text{TrkA}][\text{NGF}],\\
&R_2: && \text{TrkA:NGF} \to \text{NGF} + \text{TrkA}, && \text{rate} = k_2[\text{TrkA:NGF}],\\
&R_3: && \text{Ras:GDP} \to \text{Ras:GTP}, && \text{rate} = k_3[\text{TrkA:NGF}][\text{Ras:GDP}],\\
&R_4: && \text{Ras:GDP} \to \text{Ras:GTP}, && \text{rate} = k_4[\text{Ras:GDP}],\\
&R_5: && \text{Ras:GTP} \to \text{Ras:GDP}, && \text{rate} = k_5[\text{Ras:GTP}],\\
&R_6: && \text{Raf} \to \text{pRaf}, && \text{rate} = k_6[\text{Ras:GTP}][\text{Raf}],\\
&R_7: && \text{pRaf} \to \text{Raf}, && \text{rate} = k_7[\text{pRaf}],\\
&R_8: && \text{Mek} \to \text{pMek}, && \text{rate} = k_8[\text{pRaf}][\text{Mek}],\\
&R_9: && \text{pMek} \to \text{Mek}, && \text{rate} = k_9[\text{pMek}],\\
&R_{10}: && \text{Erk} \to \text{pErk}, && \text{rate} = k_{10}[\text{Erk}],\\
&R_{11}: && \text{pErk} \to \text{Erk}, && \text{rate} = k_{11}[\text{pErk}],
\end{aligned}
$$

and the mass conservation

$$
\begin{aligned}
[\text{TrkA}] + [\text{TrkA:NGF}] &= [\text{TrkA}]_0\\
[\text{NGF}] + [\text{TrkA:NGF}] &= [\text{NGF}]_0\\
[\text{Ras:GDP}] + [\text{Ras:GTP}] &= [\text{Ras}]_0\\
[\text{Raf}] + [\text{pRaf}] &= [\text{Raf}]_0\\
[\text{Mek}] + [\text{pMek}] &= [\text{Mek}]_0\\
[\text{Erk}] + [\text{pErk}] &= [\text{Erk}]_0.
\end{aligned}
$$

Note that we do not distinguish between c-Raf and B-Raf.

3.2.2 Reaction rate equation and parametrization

Under the assumption that the total NGF concentration is much larger than the total TrkA concentration, $[\text{NGF}]_0 \gg [\text{TrkA}]_0$, the dynamics of TrkA:NGF, Ras:GTP, pRaf, pMek and pErk can be stated as

$$
\begin{aligned}
\frac{d[\text{TrkA:NGF}]}{dt} &= k_1[\text{NGF}]_0([\text{TrkA}]_0 - [\text{TrkA:NGF}]) - k_2[\text{TrkA:NGF}]\\
\frac{d[\text{Ras:GTP}]}{dt} &= (k_3[\text{TrkA:NGF}] + k_4)([\text{Ras}]_0 - [\text{Ras:GTP}]) - k_5[\text{Ras:GTP}]\\
\frac{d[\text{pRaf}]}{dt} &= k_6[\text{Ras:GTP}]([\text{Raf}]_0 - [\text{pRaf}]) - k_7[\text{pRaf}]\\
\frac{d[\text{pMek}]}{dt} &= k_8[\text{pRaf}]([\text{Mek}]_0 - [\text{pMek}]) - k_9[\text{pMek}]\\
\frac{d[\text{pErk}]}{dt} &= k_{10}[\text{pMek}]([\text{Erk}]_0 - [\text{pErk}]) - k_{11}[\text{pErk}]\\
y &= s[\text{pErk}],
\end{aligned}
$$

with kinetic parameters $k = (k_1, k_2, k_3, k_4, k_5, k_6, k_7, k_8, k_9, k_{10}, k_{11}, [\text{TrkA}]_0, [\text{Ras}]_0, [\text{Raf}]_0, [\text{Mek}]_0, [\text{Erk}]_0, s)$. As the absolute concentrations of TrkA, Ras, Raf, Mek and Erk are unknown, this system is structurally non-identifiable. To circumvent this we reformulate the system in terms of $x_1 = k_3[\text{TrkA:NGF}]$, $x_2 = k_6[\text{Ras:GTP}]$, $x_3 = k_8[\text{pRaf}]$, $x_4 = k_{10}[\text{pMek}]$ and $x_5 = s[\text{pErk}]$, yielding the RRE model

$$
\begin{aligned}
\frac{dx_1}{dt} &= k_1[\text{NGF}]_0(k_3[\text{TrkA}]_0 - x_1) - k_2 x_1\\
\frac{dx_2}{dt} &= (x_1 + k_4)(k_6[\text{Ras}]_0 - x_2) - k_5 x_2\\
\frac{dx_3}{dt} &= x_2(k_8[\text{Raf}]_0 - x_3) - k_7 x_3\\
\frac{dx_4}{dt} &= x_3(k_{10}[\text{Mek}]_0 - x_4) - k_9 x_4\\
\frac{dx_5}{dt} &= x_4(s[\text{Erk}]_0 - x_5) - k_{11} x_5\\
y &= x_5,
\end{aligned}
$$

The solution of this ODE model merely depends on the parameters k_1, k_2, k_4, k_5, k_7, k_9 and k_{11}, and the parameter products $k_3[\mathrm{TrkA}]_0$, $k_6[\mathrm{Ras}]_0$, $k_8[\mathrm{Raf}]_0$, $k_{10}[\mathrm{Mek}]_0$ and $s[\mathrm{Erk}]_0$. Thus, we obtain the reduced vector of kinetic parameters $k = (k_1, k_2, k_4, k_5, k_7, k_9, k_{11}, k_3[\mathrm{TrkA}]_0, k_6[\mathrm{Ras}]_0, k_8[\mathrm{Raf}]_0, k_{10}[\mathrm{Mek}]_0, s[\mathrm{Erk}]_0)$.

As initial condition we use the steady state of this system for $[\mathrm{NGF}]_0 = 0$,

$$\begin{aligned}
x_1(0) &= 0\\
x_2(0) &= \frac{k_4(k_6[\mathrm{Ras}]_0)}{k_4+k_5}\\
x_3(0) &= \frac{x_2(0)(k_8[\mathrm{Raf}]_0)}{x_2(0)+k_7} = \frac{k_4(k_6[\mathrm{Ras}]_0)(k_8[\mathrm{Raf}]_0)}{k_4(k_6[\mathrm{Ras}]_0)+(k_4+k_5)k_7}\\
x_4(0) &= \frac{x_3(0)(k_{10}[\mathrm{Mek}]_0)}{x_3(0)+k_9} = \frac{k_4(k_6[\mathrm{Ras}]_0)(k_8[\mathrm{Raf}]_0)(k_{10}[\mathrm{Mek}]_0)}{k_4(k_6[\mathrm{Ras}]_0)(k_8[\mathrm{Raf}]_0)+(k_4(k_6[\mathrm{Ras}]_0)+(k_4+k_5)k_7)k_9}\\
x_5(0) &= \frac{x_4(0)(s[\mathrm{Erk}]_0)}{x_4(0)+k_{11}}\\
&= \frac{k_4(k_6[\mathrm{Ras}]_0)(k_8[\mathrm{Raf}]_0)(k_{10}[\mathrm{Mek}]_0)(s[\mathrm{Erk}]_0)}{k_4(k_6[\mathrm{Ras}]_0)(k_8[\mathrm{Raf}]_0)(k_{10}[\mathrm{Mek}]_0)+(k_4(k_6[\mathrm{Ras}]_0)(k_8[\mathrm{Raf}]_0)+(k_4(k_6[\mathrm{Ras}]_0)+(k_4+k_5)k_7)k_9)k_{11}}.
\end{aligned}$$

3.2.3 Parameter estimation

For the parameter estimation and model selection we constrained the kinetic parameter k_i to the interval $[10^{-3}, 10^3]$. The range of the kinetic parameters is smaller than for pathway model A but still six order of magnitude wide. The range had to be reduced as we encountered numerical integration problems for the ODE solvers. The subpopulation sizes w_i have been constrained to $[0, 1]$. Standard deviations of normal distributions and shape parameters of log-normal distributions were constrained to $[10^{-1}, 10^2]$ and $[10^{-1}, 10^1]$, respectively.

3.3 Pathway model C

3.3.1 Reaction network

Pathway model C accounts for the same reactions as pathway model B and an additional negative feedback from pErk to TrkA:NGF-induced Ras phosphorylation (R_3),

$$\begin{array}{lll}
R_1: & \mathrm{TrkA} + \mathrm{NGF} \to \mathrm{TrkA{:}NGF}, & \text{rate} = k_1[\mathrm{TrkA}][\mathrm{NGF}],\\
R_2: & \mathrm{TrkA{:}NGF} \to \mathrm{NGF} + \mathrm{TrkA}, & \text{rate} = k_2[\mathrm{TrkA{:}NGF}],\\
R_3: & \mathrm{Ras{:}GDP} \to \mathrm{Ras{:}GTP}, & \text{rate} = \dfrac{k_3[\mathrm{TrkA{:}NGF}][\mathrm{Ras{:}GDP}]K_3^n}{K_3^n + [\mathrm{pErk}]^n},\\
R_4: & \mathrm{Ras{:}GDP} \to \mathrm{Ras{:}GTP}, & \text{rate} = k_4[\mathrm{Ras{:}GDP}],\\
R_5: & \mathrm{Ras{:}GTP} \to \mathrm{Ras{:}GDP}, & \text{rate} = k_5[\mathrm{Ras{:}GTP}],\\
R_6: & \mathrm{Raf} \to \mathrm{pRaf}, & \text{rate} = k_6[\mathrm{Ras{:}GTP}][\mathrm{Raf}],\\
R_7: & \mathrm{pRaf} \to \mathrm{Raf}, & \text{rate} = k_7[\mathrm{pRaf}],\\
R_8: & \mathrm{Mek} \to \mathrm{pMek}, & \text{rate} = k_8[\mathrm{pRaf}][\mathrm{Mek}],\\
R_9: & \mathrm{pMek} \to \mathrm{Mek}, & \text{rate} = k_9[\mathrm{pMek}],\\
R_{10}: & \mathrm{Erk} \to \mathrm{pErk}, & \text{rate} = k_{10}[\mathrm{Erk}],\\
R_{11}: & \mathrm{pErk} \to \mathrm{Erk}, & \text{rate} = k_{11}[\mathrm{pErk}],
\end{array}$$

and the mass conservation

$$\begin{aligned}
[\mathrm{TrkA}] + [\mathrm{TrkA{:}NGF}] &= [\mathrm{TrkA}]_0\\
[\mathrm{NGF}] + [\mathrm{TrkA{:}NGF}] &= [\mathrm{NGF}]_0\\
[\mathrm{Ras{:}GDP}] + [\mathrm{Ras{:}GTP}] &= [\mathrm{Ras}]_0\\
[\mathrm{Raf}] + [\mathrm{pRaf}] &= [\mathrm{Raf}]_0\\
[\mathrm{Mek}] + [\mathrm{pMek}] &= [\mathrm{Mek}]_0\\
[\mathrm{Erk}] + [\mathrm{pErk}] &= [\mathrm{Erk}]_0.
\end{aligned}$$

Note that we do not distinguish between c-Raf and B-Raf.

3.3.2 Reaction rate equation and parametrization

Under the assumption that the total NGF concentration is much larger than the total TrkA concentration, $[\text{NGF}]_0 \gg [\text{TrkA}]_0$, the dynamics of TrkA:NGF, Ras:GTP, pRaf, pMek and pErk can be stated as

$$
\begin{aligned}
\frac{d[\text{TrkA:NGF}]}{dt} &= k_1[\text{NGF}]_0([\text{TrkA}]_0 - [\text{TrkA:NGF}]) - k_2[\text{TrkA:NGF}] \\
\frac{d[\text{Ras:GTP}]}{dt} &= \left(\frac{(k_3[\text{TrkA:NGF}]K_3^n}{K_3^n + [\text{pErk}]^n} + k_4\right)([\text{Ras}]_0 - [\text{Ras:GTP}]) - k_5[\text{Ras:GTP}] \\
\frac{d[\text{pRaf}]}{dt} &= k_6[\text{Ras:GTP}]([\text{Raf}]_0 - [\text{pRaf}]) - k_7[\text{pRaf}] \\
\frac{d[\text{pMek}]}{dt} &= k_8[\text{pRaf}]([\text{Mek}]_0 - [\text{pMek}]) - k_9[\text{pMek}] \\
\frac{d[\text{pErk}]}{dt} &= k_{10}[\text{pMek}]([\text{Erk}]_0 - [\text{pErk}]) - k_{11}[\text{pErk}] \\
y &= s[\text{pErk}],
\end{aligned}
$$

with kinetic parameters

$$k = (k_1, k_2, k_3, K_3, k_4, k_5, k_6, k_7, k_8, k_9, k_{10}, k_{11}, [\text{TrkA}]_0, [\text{Ras}]_0, [\text{Raf}]_0, [\text{Mek}]_0, [\text{Erk}]_0, s, K_3, n).$$

As the absolute concentrations of TrkA, Ras, Raf, Mek and Erk are unknown, this system is structurally non-identifiable. To circumvent this we reformulate the system in terms of $x_1 = k_3[\text{TrkA:NGF}]$, $x_2 = k_6[\text{Ras:GTP}]$, $x_3 = k_8[\text{pRaf}]$, $x_4 = k_{10}[\text{pMek}]$ and $x_5 = s[\text{pErk}]$, yielding the RRE model

$$
\begin{aligned}
\frac{dx_1}{dt} &= k_1[\text{NGF}]_0(k_3[\text{TrkA}]_0 - x_1) - k_2x_1 \\
\frac{dx_2}{dt} &= \left(\frac{x_1(sK_3)^n}{(sK_3)^n + x_5^n} + k_4\right)(k_6[\text{Ras}]_0 - x_2) - k_5x_2 \\
\frac{dx_3}{dt} &= x_2(k_8[\text{Raf}]_0 - x_3) - k_7x_3 \\
\frac{dx_4}{dt} &= x_3(k_{10}[\text{Mek}]_0 - x_4) - k_9x_4 \\
\frac{dx_5}{dt} &= x_4(s[\text{Erk}]_0 - x_5) - k_{11}x_5 \\
y &= x_5,
\end{aligned}
$$

The solution of this ODE model merely depends on the parameters k_1, k_2, k_4, k_5, k_7, k_9 and k_{11}, and the parameter products $k_3[\text{TrkA}]_0$, sK_3, $k_6[\text{Ras}]_0$, $k_8[\text{Raf}]_0$, $k_{10}[\text{Mek}]_0$ and $s[\text{Erk}]_0$. Thus, we obtain the reduced vector of kinetic parameters

$$k = (k_1, k_2, k_4, k_5, k_7, k_9, k_{11}, k_3[\text{TrkA}]_0, k_6[\text{Ras}]_0, k_8[\text{Raf}]_0, k_{10}[\text{Mek}]_0, s[\text{Erk}]_0, sK_3, n).$$

As initial condition we use the steady state of this system for $[\text{NGF}]_0 = 0$, which is equivalent to the initial condition for pathway model B.

3.3.3 Parameter estimation

For the parameter estimation and model selection we constrained the kinetic parameter k_i to the interval $[10^{-3}, 10^3]$. The range of the kinetic parameters is smaller than for pathway model A but still six order of magnitude wide. The range had to be reduced as we encountered numerical integration problems for the ODE solvers. The subpopulation sizes w_i have been constrained to $[0, 1]$. Standard deviations of normal distributions and shape parameters of log-normal distributions were constrained to $[10^{-1}, 10^2]$ and $[10^{-1}, 10^1]$, respectively.

Pathway model C possesses a negative feedback loop and can oscillate. It turned out that several runs of the local optimiser converged to parameters for which the system exhibits very fast oscillations. As very fast oscillations have not been observed experimentally, we filter the parameter for which the system oscillates heavily. More precisely, we excluded parameters for which the simulated trajectory for 1 ng/ml NGF exhibit more than five peaks in the time interval from 0 to 60 min.

4 Implementation

The simulation, estimation and uncertainty analysis of ODE-MMs has been implemented in MATLAB R2103a (for MAC). The local optimisation was performed with the MATLAB routine `fmincon.m`. For the profile likelihood calculation an in-house code was used. For the simulation we employed SUNDIALSTB, the MATLAB interface to Suite of Nonlinear and Differential/Algebraic Equation Solvers (SUNDIALS) [4].

All routines are available as supporting material, including the m-files used to prepare the plots.

References

[1] Andres C, Meyer S, Dina OA, Levine JD, Hucho T (2010) Quantitative automated microscopy (QuAM) elucidates growth factor specific signalling in pain sensitization. Molecular Pain 6: 1–16.

[2] Raue A, Kreutz C, Maiwald T, Bachmann J, Schilling M, et al. (2009) Structural and practical identifiability analysis of partially observed dynamical models by exploiting the profile likelihood. Bioinf 25: 1923–1929.

[3] Schwarz G (1978) Estimating the dimension of a model. Ann Statist 6: 461–464.

[4] Hindmarsh AC, Brown PN, Grant KE, Lee SL, Serban R, et al. (2005) SUNDIALS: Suite of Nonlinear and Differential/Algebraic Equation Solvers. ACM Trans on Math Softw 31: 363–396.

Supplement Table 1: **Parameter estimation and model selection results for NGF-induced Erk1/2 signalling.** For each biological hypothesis (H1: no subpopulations; H2: different levels of total Erk, $[\mathrm{Erk}]_0$; H3: different levels of total TrkA, $[\mathrm{TrkA}]_0$) three models, differing in the distribution assumption (normal vs. log-normal) and the ODE constrained properties (subpopulation mean vs. subpopulation median), have been specified and fitted to the experimental data using multi-start local optimisation (accuracy: 10 digits). This analysis has been performed for all pathway models.

Pathway model A (simple)

	m	distribution	ODE const.	variability	# par.	$\ell(\theta^*)(10^4)$	BIC (10^4)	rank	Δ_{BIC}	decision
$\mathcal{M}_{\mathrm{H1,1}}$	1	normal	mean	-	17	-5.2890	10.5955	9	>10	rejected
$\mathcal{M}_{\mathrm{H1,2}}$	1	log-normal	mean	-	17	-3.7659	7.5495	6	>10	rejected
$\mathcal{M}_{\mathrm{H1,3}}$	1	log-normal	median	-	17	-3.7556	7.5288	5	>10	rejected
$\mathcal{M}_{\mathrm{H2,1}}$	2	normal	mean	$[\mathrm{Erk}]_0$	30	-4.0348	8.1006	8	>10	rejected
$\mathcal{M}_{\mathrm{H2,2}}$	2	log-normal	mean	$[\mathrm{Erk}]_0$	30	-3.6482	7.3274	4	>10	rejected
$\mathcal{M}_{\mathrm{H2,3}}$	2	log-normal	median	$[\mathrm{Erk}]_0$	30	-3.6262	7.2835	3	>10	rejected
$\mathcal{M}_{\mathrm{H3,1}}$	2	normal	mean	$[\mathrm{TrkA}]_0$	30	-3.9846	8.0002	7	>10	rejected
$\mathcal{M}_{\mathrm{H3,2}}$	2	log-normal	mean	$[\mathrm{TrkA}]_0$	30	-3.5847	7.2003	2	2.189	not rejected
$\mathcal{M}_{\mathrm{H3,3}}$	2	log-normal	median	$[\mathrm{TrkA}]_0$	30	-3.5846	7.2001	1	0	optimal

Pathway model B (cascade)

	m	distribution	ODE const.	variability	# par.	$\ell(\theta^*)(10^4)$	BIC (10^4)	rank	Δ_{BIC}	decision
$\mathcal{M}_{\mathrm{H1,1}}$	1	normal	mean	-	23	-5.2888	10.6013	9	>10	rejected
$\mathcal{M}_{\mathrm{H1,2}}$	1	log-normal	mean	-	23	-3.7655	7.5548	6	>10	rejected
$\mathcal{M}_{\mathrm{H1,3}}$	1	log-normal	median	-	23	-3.7555	7.5348	5	>10	rejected
$\mathcal{M}_{\mathrm{H2,1}}$	2	normal	mean	$[\mathrm{Erk}]_0$	36	-4.0347	8.1067	8	>10	rejected
$\mathcal{M}_{\mathrm{H2,2}}$	2	log-normal	mean	$[\mathrm{Erk}]_0$	36	-3.6380	7.3133	4	>10	rejected
$\mathcal{M}_{\mathrm{H2,3}}$	2	log-normal	median	$[\mathrm{Erk}]_0$	36	-3.6261	7.2894	3	>10	rejected
$\mathcal{M}_{\mathrm{H3,1}}$	2	normal	mean	$[\mathrm{TrkA}]_0$	36	-3.9767	7.9906	7	>10	rejected
$\mathcal{M}_{\mathrm{H3,2}}$	2	log-normal	mean	$[\mathrm{TrkA}]_0$	36	-3.5853	7.2079	2	>10	rejected
$\mathcal{M}_{\mathrm{H3,3}}$	2	log-normal	median	$[\mathrm{TrkA}]_0$	36	-3.5797	7.1965	1	0	optimal

Pathway model C (cascade & feedback)

	m	distribution	ODE const.	variability	# par.	$\ell(\theta^*)(10^4)$	BIC (10^4)	rank	Δ_{BIC}	decision
$\mathcal{M}_{\mathrm{H1,1}}$	1	normal	mean	-	25	-5.2809	10.5876	9	>10	rejected
$\mathcal{M}_{\mathrm{H1,2}}$	1	log-normal	mean	-	25	-3.7461	7.5181	6	>10	rejected
$\mathcal{M}_{\mathrm{H1,3}}$	1	log-normal	median	-	25	-3.7432	7.5123	5	>10	rejected
$\mathcal{M}_{\mathrm{H2,1}}$	2	normal	mean	$[\mathrm{Erk}]_0$	38	-4.0347	8.1087	8	>10	rejected
$\mathcal{M}_{\mathrm{H2,2}}$	2	log-normal	mean	$[\mathrm{Erk}]_0$	38	-3.6380	7.3154	4	>10	rejected
$\mathcal{M}_{\mathrm{H2,3}}$	2	log-normal	median	$[\mathrm{Erk}]_0$	38	-3.5849	7.2091	3	>10	rejected
$\mathcal{M}_{\mathrm{H3,1}}$	2	normal	mean	$[\mathrm{TrkA}]_0$	38	-3.9676	7.9745	7	>10	rejected
$\mathcal{M}_{\mathrm{H3,2}}$	2	log-normal	mean	$[\mathrm{TrkA}]_0$	38	-3.5743	7.1878	2	>10	rejected
$\mathcal{M}_{\mathrm{H3,3}}$	2	log-normal	median	$[\mathrm{TrkA}]_0$	38	-3.5719	7.1830	1	0	optimal

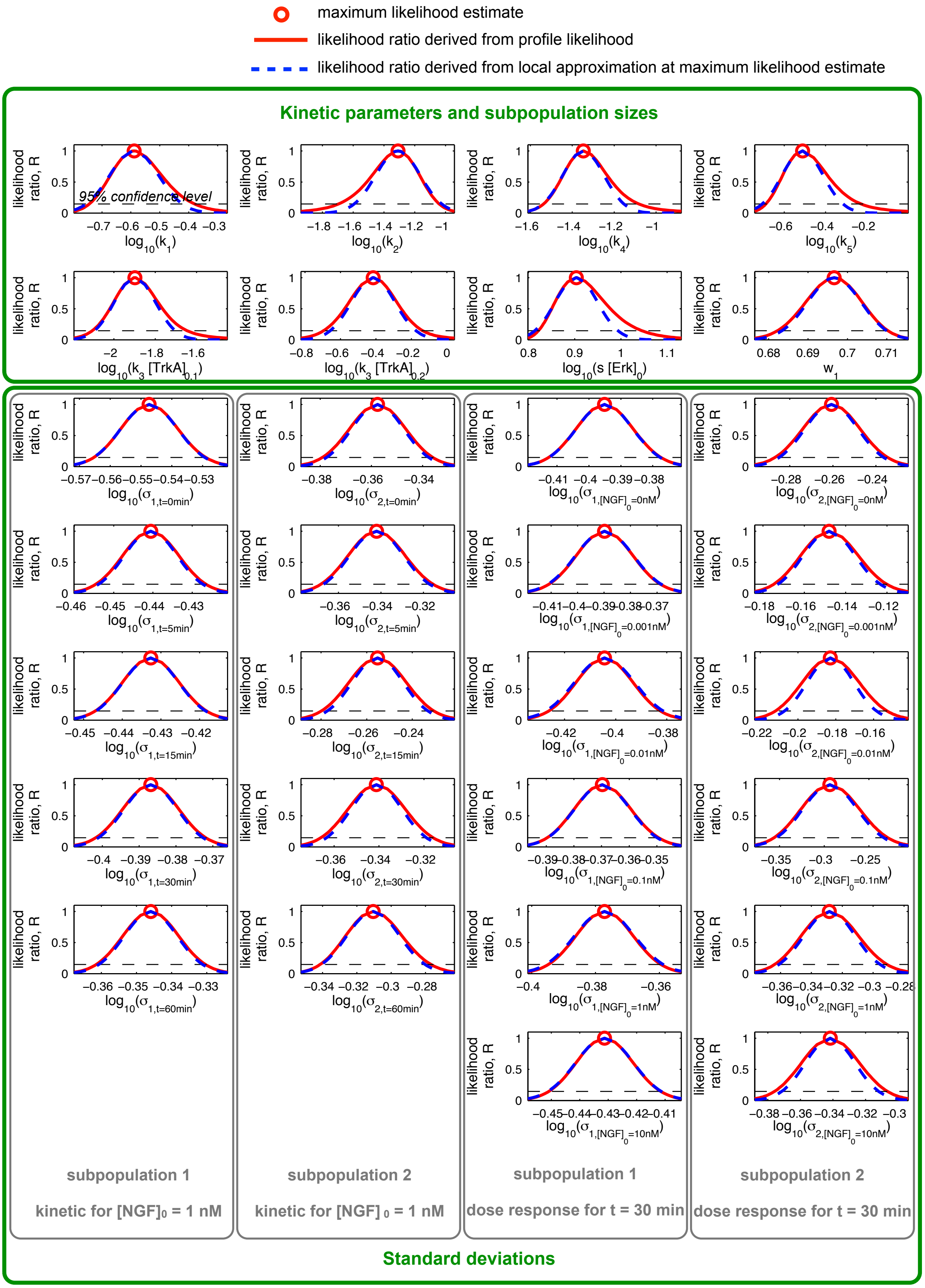

Supplement Figure 1: **Uncertainty analysis of $\mathcal{M}_{\mathbf{H3,2}}$ with Pathway model A.** For all unknown parameters (kinetic parameters, subpopulation sizes and standard deviations) the maximum likelihood estimates and the likelihood ratio profiles are depicted. The profile for the likelihood ratio profiles have been computed using the methods of profile likelihoods [2] and using a quadratic approximation at the maximum likelihood estimate.

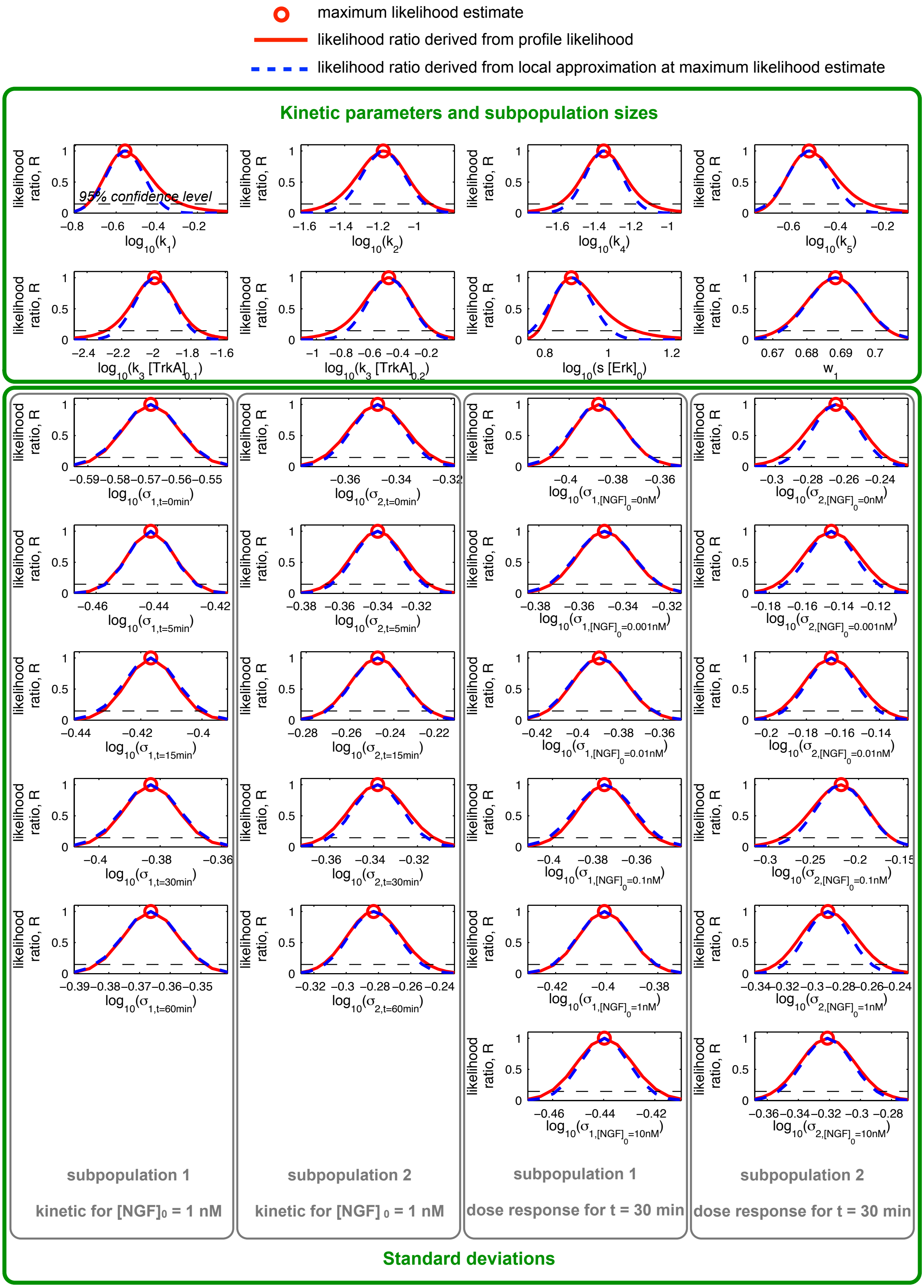

Supplement Figure 2: **Uncertainty analysis of $\mathcal{M}_{\mathbf{H3,2}}$ with Pathway model A.** For all unknown parameters (kinetic parameters, subpopulation sizes and standard deviations) the maximum likelihood estimates and the likelihood ratio profiles are depicted. The profile for the likelihood ratio profiles have been computed using the methods of profile likelihoods [2] and using a quadratic approximation at the maximum likelihood estimate.

Supplement Table 2: **Parameter estimates and confidence intervals for NGF induced Erk1/2 signalling, Pathway model A: Reaction rate constants, protein concentrations and subpopulation sizes.** Maximum likelihood estimates followed by the 99% confidence intervals (in brackets) computed using the method of profile likelihoods. If lower or upper bounds for the confidence intervals are not contained in the interval $[-6.0, +6.0]$, we write < -6.0 or $> +6.0$, respectively. The profile likelihood derived confidence intervals indicate that several parameters are practical non-identifiable for hypothesis 1 (H1: no subpopulations) and 2 (H2: different levels of total Erk, $[\text{Erk}]_0$), as well as for model $\mathcal{M}_{\text{H3},1}$ describing hypothesis 3 (H3: different levels of total TrkA, $[\text{TrkA}]_0$). For the models $\mathcal{M}_{\text{H3},2}$ and $\mathcal{M}_{\text{H3},3}$ describing hypothesis 3 all model parameters are identifiable.

	weights w		$\log_{10}(k_1)$	$\log_{10}(k_2)$	$\log_{10}(k_4)$
	w_1	$w_2(= 1 - w_1)$			
$\mathcal{M}_{\text{H1},1}$	1		−0.589 (< −6.0, +0.757)	−0.935 (−2.050, > +6.0)	−1.191 (−2.389, +0.668)
$\mathcal{M}_{\text{H1},2}$	1		−0.590 (< −6.0, +0.064)	−0.955 (−1.519, −0.304)	−1.211 (−1.849, −0.880)
$\mathcal{M}_{\text{H1},3}$	1		−0.535 (< −6.0, +0.164)	−0.798 (−1.516, +1.025)	−1.010 (−1.765, −0.391)
$\mathcal{M}_{\text{H2},1}$	+0.719 (+0.709, +0.729)	+0.281 (+0.271, +0.291)	−0.411 (−0.673, +2.923)	−0.864 (−1.193, +2.634)	−4.743 (< −6.0, +3.255)
$\mathcal{M}_{\text{H2},2}$	+0.805 (+0.756, +0.853)	+0.195 (+0.147, +0.244)	−1.577 (< −6.0, > +6.0)	+4.445 (−0.624, > +6.0)	−1.448 (−1.713, > +6.0)
$\mathcal{M}_{\text{H2},3}$	+0.360 (+0.336, +0.385)	+0.640 (+0.615, +0.664)	−0.467 (−0.468, −0.466)	−0.764 (−1.312, +0.394)	−0.902 (−1.540, −0.490)
$\mathcal{M}_{\text{H3},1}$	+0.687 (+0.302, +0.324)	+0.313 (+0.676, +0.698)	+0.891 (+0.456, > +6.0)	−0.644 (−1.061, > +6.0)	−5.330 (< −6.0, −1.851)
$\mathcal{M}_{\text{H3},2}$	+0.697 (+0.678, +0.714)	+0.303 (+0.286, +0.322)	−0.589 (−0.789, −0.284)	−1.309 (−1.903, −0.956)	−1.347 (−1.603, −0.911)
$\mathcal{M}_{\text{H3},3}$	+0.688 (+0.665, +0.709)	+0.312 (+0.291, +0.335)	−0.556 (−0.795, −0.099)	−1.190 (−1.616, −0.821)	−1.368 (−1.751, −0.976)

	$\log_{10}(k_3[\text{TrkA}]_0)$		$\log_{10}(k_5)$	$\log_{10}(s[\text{Erk}]_0)$	
	$\log_{10}(k_3[\text{TrkA}]_{0,1})$	$\log_{10}(k_3[\text{TrkA}]_{0,2})$		$\log_{10}(s[\text{Erk}]_{0,1})$	$\log_{10}(s[\text{Erk}]_{0,2})$
$\mathcal{M}_{\text{H1},1}$	−0.407 (< −6.0, > +6.0)		−0.920 (−2.317, > +6.0)	+0.474 (+0.352, > +6.0)	
$\mathcal{M}_{\text{H1},2}$	−0.406 (< −6.0, > +6.0)		−1.005 (−1.801, −0.231)	+0.444 (+0.350, > +6.0)	
$\mathcal{M}_{\text{H1},3}$	−0.387 (< −6.0, > +6.0)		−0.916 (−1.877, +1.017)	+0.341 (+0.232, > +6.0)	
$\mathcal{M}_{\text{H2},1}$	−5.103 (< −6.0, > +6.0)		−0.230 (−0.511, +1.207)	+4.472 (+0.087, > +6.0)	+4.930 (+0.552, > +6.0)
$\mathcal{M}_{\text{H2},2}$	+5.366 (< −6.0, > +6.0)		−1.736 (−2.025, > +6.0)	+0.151 (+0.103, > +6.0)	+0.706 (+0.658, > +6.0)
$\mathcal{M}_{\text{H2},3}$	−0.433 (< −6.0, > +6.0)		−0.818 (−1.713, +0.418)	+0.192 (+0.063, > +6.0)	+0.429 (+0.300, > +6.0)
$\mathcal{M}_{\text{H3},1}$	−5.971 (< −6.0, −1.186)	−4.792 (< −6.0, −2.450)	−0.562 (−0.620, −0.502)	+4.745 (+1.198, > +6.0)	
$\mathcal{M}_{\text{H3},2}$	−1.899 (−2.182, −1.487)	−0.416 (−0.803, +0.009)	−0.511 (−0.736, −0.048)	+0.904 (+0.802, +1.118)	
$\mathcal{M}_{\text{H3},3}$	−2.009 (−2.449, −1.618)	−0.489 (−1.067, −0.049)	−0.526 (−0.744, −0.134)	+0.884 (+0.754, +1.209)	

Supplement Table 3: **Parameter estimates and confidence intervals for NGF induced Erk1/2 signalling, Pathway model A: Standard deviations for kinetic for $[\mathbf{NGF}]_0 = 1$ nM.** Maximum likelihood estimates followed by the 99% confidence intervals (in brackets) computed using the method of profile likelihoods. The profile likelihood derived confidence intervals indicate that all standard deviations are practical identifiable.

Subpopulation 1

	$\log_{10}(\sigma_{1,t=0\,\mathrm{min}})$	$\log_{10}(\sigma_{1,t=5\,\mathrm{min}})$	$\log_{10}(\sigma_{1,t=15\,\mathrm{min}})$	$\log_{10}(\sigma_{1,t=30\,\mathrm{min}})$	$\log_{10}(\sigma_{1,t=60\,\mathrm{min}})$
$\mathcal{M}_{\mathrm{H1},1}$	−0.405 (−0.419, −0.392)	+0.141 (+0.129, +0.153)	+0.408 (+0.395, +0.421)	+0.301 (+0.288, +0.314)	+0.363 (+0.350, +0.377)
$\mathcal{M}_{\mathrm{H1},2}$	−0.454 (−0.468, −0.440)	−0.207 (−0.219, −0.195)	−0.156 (−0.168, −0.144)	−0.152 (−0.164, −0.140)	−0.101 (−0.113, −0.088)
$\mathcal{M}_{\mathrm{H1},3}$	−0.473 (−0.487, −0.460)	−0.208 (−0.220, −0.196)	−0.140 (−0.153, −0.127)	−0.146 (−0.159, −0.133)	−0.102 (−0.116, −0.088)
$\mathcal{M}_{\mathrm{H2},1}$	−0.616 (−0.636, −0.597)	−0.412 (−0.437, −0.387)	−0.361 (−0.386, −0.335)	−0.343 (−0.371, −0.316)	−0.351 (−0.378, −0.324)
$\mathcal{M}_{\mathrm{H2},2}$	−0.559 (−0.575, −0.542)	−0.386 (−0.404, −0.367)	−0.396 (−0.415, −0.377)	−0.341 (−0.360, −0.323)	−0.297 (−0.318, −0.277)
$\mathcal{M}_{\mathrm{H2},3}$	−0.647 (−0.675, −0.619)	−0.564 (−0.601, −0.528)	−0.569 (−0.610, −0.529)	−0.500 (−0.545, −0.455)	−0.498 (−0.546, −0.450)
$\mathcal{M}_{\mathrm{H3},1}$	−0.641 (−0.662, −0.620)	−0.431 (−0.454, −0.409)	−0.367 (−0.391, −0.343)	−0.358 (−0.383, −0.333)	−0.383 (−0.407, −0.359)
$\mathcal{M}_{\mathrm{H3},2}$	−0.547 (−0.571, −0.523)	−0.441 (−0.459, −0.422)	−0.433 (−0.451, −0.414)	−0.387 (−0.406, −0.367)	−0.346 (−0.366, −0.325)
$\mathcal{M}_{\mathrm{H3},3}$	−0.569 (−0.593, −0.545)	−0.442 (−0.462, −0.422)	−0.417 (−0.438, −0.395)	−0.383 (−0.406, −0.360)	−0.366 (−0.390, −0.343)

Subpopulation 2

	$\log_{10}(\sigma_{2,t=0\,\mathrm{min}})$	$\log_{10}(\sigma_{2,t=5\,\mathrm{min}})$	$\log_{10}(\sigma_{2,t=15\,\mathrm{min}})$	$\log_{10}(\sigma_{2,t=30\,\mathrm{min}})$	$\log_{10}(\sigma_{2,t=60\,\mathrm{min}})$
$\mathcal{M}_{\mathrm{H1},1}$	–	–	–	–	–
$\mathcal{M}_{\mathrm{H1},2}$	–	–	–	–	–
$\mathcal{M}_{\mathrm{H1},3}$	–	–	–	–	–
$\mathcal{M}_{\mathrm{H2},1}$	+0.096 (+0.048, +0.145)	+0.209 (+0.184, +0.235)	+0.551 (+0.526, +0.577)	+0.381 (+0.357, +0.406)	+0.479 (+0.452, +0.506)
$\mathcal{M}_{\mathrm{H2},2}$	+0.024 (−0.012, +0.060)	−0.413 (−0.452, −0.372)	−0.327 (−0.360, −0.293)	−0.402 (−0.435, −0.369)	−0.374 (−0.409, −0.337)
$\mathcal{M}_{\mathrm{H2},3}$	−0.523 (−0.545, −0.499)	−0.181 (−0.198, −0.164)	−0.104 (−0.122, −0.086)	−0.120 (−0.138, −0.103)	−0.059 (−0.077, −0.040)
$\mathcal{M}_{\mathrm{H3},1}$	−0.181 (−0.210, −0.151)	+0.207 (+0.185, +0.230)	+0.528 (+0.505, +0.551)	+0.363 (+0.341, +0.385)	+0.453 (+0.429, +0.477)
$\mathcal{M}_{\mathrm{H3},2}$	−0.358 (−0.386, −0.329)	−0.342 (−0.376, −0.307)	−0.255 (−0.288, −0.223)	−0.341 (−0.374, −0.308)	−0.310 (−0.351, −0.265)
$\mathcal{M}_{\mathrm{H3},3}$	−0.349 (−0.377, −0.319)	−0.342 (−0.377, −0.307)	−0.247 (−0.280, −0.215)	−0.338 (−0.371, −0.304)	−0.283 (−0.325, −0.238)

Supplement Table 4: **Parameter estimates and confidence intervals for NGF induced Erk1/2 signalling, Pathway model A: Standard deviations for dose response for $t = 30$ min.** Maximum likelihood estimates followed by the 99% confidence intervals (in brackets) computed using the method of profile likelihoods. The profile likelihood derived confidence intervals indicate that all standard deviations are practical identifiable.

Subpopulation 1

	$\log_{10}(\sigma_{1,[\mathrm{NGF}]_0=0\,\mathrm{nM}})$	$\log_{10}(\sigma_{1,[\mathrm{NGF}]_0=0.001\,\mathrm{nM}})$	$\log_{10}(\sigma_{1,[\mathrm{NGF}]_0=0.01\,\mathrm{nM}})$	$\log_{10}(\sigma_{1,[\mathrm{NGF}]_0=0.1\,\mathrm{nM}})$	$\log_{10}(\sigma_{1,[\mathrm{NGF}]_0=1\,\mathrm{nM}})$	$\log_{10}(\sigma_{1,[\mathrm{NGF}]_0=10\,\mathrm{nM}})$
$\mathcal{M}_{\mathrm{H1,1}}$	−0.203 (−0.218, −0.188)	+0.129 (+0.111, +0.147)	−0.050 (−0.069, −0.031)	+0.054 (+0.033, +0.076)	+0.291 (+0.274, +0.308)	+0.290 (+0.272, +0.307)
$\mathcal{M}_{\mathrm{H1,2}}$	−0.345 (−0.359, −0.330)	−0.292 (−0.309, −0.275)	−0.290 (−0.308, −0.272)	−0.192 (−0.215, −0.168)	−0.120 (−0.135, −0.104)	−0.153 (−0.170, −0.136)
$\mathcal{M}_{\mathrm{H1,3}}$	−0.342 (−0.357, −0.327)	−0.265 (−0.283, −0.247)	−0.272 (−0.291, −0.253)	−0.219 (−0.240, −0.197)	−0.135 (−0.152, −0.118)	−0.162 (−0.179, −0.144)
$\mathcal{M}_{\mathrm{H2,1}}$	−0.504 (−0.530, −0.479)	−0.396 (−0.434, −0.359)	−0.482 (−0.512, −0.452)	−0.456 (−0.491, −0.419)	−0.369 (−0.398, −0.340)	−0.368 (−0.400, −0.335)
$\mathcal{M}_{\mathrm{H2,2}}$	−0.406 (−0.424, −0.387)	−0.374 (−0.399, −0.350)	−0.398 (−0.422, −0.372)	−0.351 (−0.379, −0.323)	−0.299 (−0.324, −0.273)	−0.341 (−0.369, −0.313)
$\mathcal{M}_{\mathrm{H2,3}}$	−0.594 (−0.635, −0.555)	−0.568 (−0.628, −0.507)	−0.553 (−0.609, −0.498)	−0.549 (−0.608, −0.491)	−0.492 (−0.537, −0.447)	−0.546 (−0.597, −0.496)
$\mathcal{M}_{\mathrm{H3,1}}$	−0.499 (−0.523, −0.475)	−0.392 (−0.426, −0.358)	−0.471 (−0.501, −0.441)	−0.382 (−0.413, −0.351)	−0.395 (−0.422, −0.367)	−0.408 (−0.437, −0.378)
$\mathcal{M}_{\mathrm{H3,2}}$	−0.395 (−0.418, −0.372)	−0.390 (−0.417, −0.362)	−0.404 (−0.433, −0.376)	−0.370 (−0.396, −0.343)	−0.377 (−0.400, −0.354)	−0.431 (−0.457, −0.405)
$\mathcal{M}_{\mathrm{H3,3}}$	−0.388 (−0.415, −0.358)	−0.350 (−0.382, −0.316)	−0.391 (−0.425, −0.357)	−0.376 (−0.407, −0.345)	−0.401 (−0.427, −0.374)	−0.440 (−0.468, −0.411)

Subpopulation 2

	$\log_{10}(\sigma_{2,[\mathrm{NGF}]_0=0\,\mathrm{nM}})$	$\log_{10}(\sigma_{2,[\mathrm{NGF}]_0=0.001\,\mathrm{nM}})$	$\log_{10}(\sigma_{2,[\mathrm{NGF}]_0=0.01\,\mathrm{nM}})$	$\log_{10}(\sigma_{2,[\mathrm{NGF}]_0=0.1\,\mathrm{nM}})$	$\log_{10}(\sigma_{2,[\mathrm{NGF}]_0=1\,\mathrm{nM}})$	$\log_{10}(\sigma_{2,[\mathrm{NGF}]_0=10\,\mathrm{nM}})$
$\mathcal{M}_{\mathrm{H1,1}}$	–	–	–	–	–	–
$\mathcal{M}_{\mathrm{H1,2}}$	–	–	–	–	–	–
$\mathcal{M}_{\mathrm{H1,3}}$	–	–	–	–	–	–
$\mathcal{M}_{\mathrm{H2,1}}$	+0.072 (+0.029, +0.117)	+0.377 (+0.336, +0.422)	+0.084 (+0.044, +0.127)	+0.162 (+0.119, +0.207)	+0.394 (+0.364, +0.427)	+0.369 (+0.338, +0.402)
$\mathcal{M}_{\mathrm{H2,2}}$	−0.015 (−0.062, +0.030)	−0.182 (−0.254, −0.111)	−0.222 (−0.320, −0.127)	−0.364 (−0.453, −0.246)	−0.373 (−0.426, −0.314)	−0.404 (−0.454, −0.350)
$\mathcal{M}_{\mathrm{H2,3}}$	−0.361 (−0.385, −0.337)	−0.288 (−0.311, −0.264)	−0.281 (−0.306, −0.256)	−0.190 (−0.218, −0.160)	−0.093 (−0.116, −0.070)	−0.125 (−0.148, −0.101)
$\mathcal{M}_{\mathrm{H3,1}}$	+0.048 (+0.016, +0.082)	+0.427 (+0.386, +0.473)	+0.102 (+0.064, +0.144)	+0.208 (+0.157, +0.260)	+0.372 (+0.342, +0.404)	+0.350 (+0.320, +0.381)
$\mathcal{M}_{\mathrm{H3,2}}$	−0.261 (−0.295, −0.226)	−0.148 (−0.183, −0.111)	−0.183 (−0.221, −0.143)	−0.294 (−0.375, −0.209)	−0.328 (−0.375, −0.279)	−0.342 (−0.386, −0.295)
$\mathcal{M}_{\mathrm{H3,3}}$	−0.266 (−0.310, −0.229)	−0.146 (−0.186, −0.106)	−0.166 (−0.205, −0.127)	−0.219 (−0.309, −0.147)	−0.291 (−0.340, −0.239)	−0.321 (−0.367, −0.275)
