# Supplementary material for: ODE Constrained Mixture Modelling: A Method for Unraveling Subpopulation Structures and Dynamics
Source: PLoS Comput Biol. 2014 Jul 3;10(7):e1003686. doi: 10.1371/journal.pcbi.1003686 (PMC4081021; doi:10.1371/journal.pcbi.1003686)
Supplement: Code S1 — MATLAB code used for ODE constrained mixture modelling. This zip-file contains the MATLAB code for the simulation example (conversion process) and the application example (NGF-induced Erk1/2 phosphorylation) presented in the paper. We provide implementations for the models, the parameter estimation, the uncertainty analysis and the model selection. In addition to the implementation, also all data and result files (.mat) are included. (ZIP) [file pcbi.1003686.s001.zip › ODEMM_implementation_v01/README.txt]

%%%%%%%%%%%%%%%%%%%%%%%%%%%%
%%                                                                             %%
%%    Tool for ODE constrained mixture modelling    %%
%%                                                                             %%
%%%%%%%%%%%%%%%%%%%%%%%%%%%%

1) Description:

This is a collection of MATLAB algorithm for ODE constrained
mixture modelling and provides an implementation of the examples
presented in the manuscript: 

[1] J. Hasenauer, C. Andres, Tim Hucho and F. Theis.
     ODE constrained mixture modelling: A method for
     unraveling subpopulation structures and dynamics
     submitted to PLoS Computational Biology

Note: The software is not intended as a complete toolbox, but should
only provide a first simple, easily extendable implementation of the
methods.


%%%%%%%%%%%%%%%%%%%%%%%%%%%%

2) Installation:

To employ the functionality of the tools include the place were it is
located into your MATLAB path. Make sure that you have the MATLAB
statistical toolbox and MATLAB optimisation toolbox are available.
Furthermore the SUNDIALS package CVodes and SUNDIALSTB
have to be installed. These toolboxes are used for an efficient simulation.


%%%%%%%%%%%%%%%%%%%%%%%%%%%%

3) Content and functionality:

For an illustration of the functionality we refer to the example
     ./conversion_reaction/scenario_1___low_variability
     ./conversion_reaction/scenario_2___high_variability
     ./NGF_induced_Erk_phosphorylation/pathway_model_A
     ./NGF_induced_Erk_phosphorylation/pathway_model_B
     ./NGF_induced_Erk_phosphorylation/pathway_model_C

These example also contain the results presented in the paper and 
routines which generate the respective plots:
     plots_for_paper.m
     plots_for_paper__model_comparison.m


Furthermore, the measurement data for the NGF signalling
pathway are provided in 
     ./NGF_induced_Erk_phosphorylation/pathway_model_A/project/data
For a consistent way to load these data we refer to the m-file
fitting__2logn_median_TrkA0.m in the subfolder
    ./NGF_induced_Erk_phosphorylation/pathway_model_A

%%%%%%%%%%%%%%%%%%%%%%%%%%%%

4) Contacts:

This tool has been developed by Jan Hasenauer. If you need support or
if you have any recommendations how the tools can be improved, please
do not hesitate to contact me:

Jan Hasenauer
Institute of Computational Biology
Helmholtz Center Munich
Email: jan.hasenauer@helmholtz-muenchen.de
